# Why Does the Optimal Tuning Method of the Range Separation Parameter of a Long-Range Corrected Density Functional Fail in Intramolecular Charge Transfer Excitation Calculations?

**DOI:** 10.3390/molecules29184423

**Published:** 2024-09-18

**Authors:** Han-Seok Bae, Dae-Hwan Ahn, Jong-Won Song

**Affiliations:** Department of Chemistry Education, Daegu University, Gyeongsan-si 113-8656, Republic of Korea; 16336@naver.com (H.-S.B.); qmdleoghks1@daegu.ac.kr (D.-H.A.)

**Keywords:** range separation, DFT, LC-DFT, charge transfer, polyene, optimal tuning, intramolecular CT

## Abstract

We performed intra- and intermolecular charge transfer (CT) excitation energy calculations of (a) conjugated carbon chain [H_2_N–(CH=CH)*_n_*–X] and (b) its equidistant H_2_NH∙∙∙H*X* (*n* = 2~8) with various electron acceptors (*X* = NH_2_, OH, Cl, CHO, CN, and NO_2_) using EOM-CCSD, time-dependent (TD) Hartree–Fock (HF) and various density functional theory (DFT) functionals, such as BLYP, B3LYP, long-range corrected (LC) DFT, and LC-DFT with an optimally tuned (OT) range separation parameter (*µ*) using Koopman’s theorem to investigate the effect of the electron-withdrawing (or -donating) strength of end-capped functional group (*X*) and CT distance (*R*) on intra- and intermolecular CT excitation energies. As the electron-withdrawing strength of *X* increases, both intra- and intermolecular CT excitation energies tend to decrease, since energy gaps between orbitals corresponding to CT excitations (e.g., HOMO and LUMO) decrease. However, the effect of the electron-withdrawing group on intramolecular CT excitation energy is negligible (at most 0.5 eV). OT-LC-DFT shows accurate intermolecular CT excitation energy, but worse results in intramolecular CT excitation energy than LC-DFT with the default *µ* value (0.47). Therefore, we conclude that the optimal tuning method is not effective in predicting intramolecular CT excitation energy. While intermolecular CT excitation energy has excitonic binding energy with asymptotic behavior to CT distance that is not affected by the choice of range separation parameter, intramolecular CT excitation energy is affected by orbital relaxation energy, which strongly depends on the choice of range separation parameter, which makes the OT method of range separation parameter ineffective in predicting intramolecular CT excitation energy as well as local excitation with high accuracy.

## 1. Introduction

Charge transfer (CT) is a core photochemical mechanism of materials applied in various fields from biology to green energy, such as photosynthesis, photovoltaic cells, and solar cells. Thus, it is necessary to accurately predict inter- and intramolecular CT excitation energies in many fields [1,2,3,4,5,6,7]. Accordingly, a time-dependent (TD) scheme using density functional theory [8] (DFT) functionals can be applied to the calculations of the excited state of large systems at a relatively low computational cost. However, conventional DFT functionals, such as local density approximation (LDA), generalized gradient approximation (GGA), and global hybrid functionals, appear to have systematic errors in calculating both excitonic binding and orbital energies due to local characteristics of the exchange functional. As a result, since CT excitation energy cannot be accurately calculated, many related studies have been conducted. Studies to predict high-accuracy CT excitation energy using DFT combined with a long-range exchange correction [9,10] (LC) scheme, with its range separation parameter (*µ*) optimized to Koopmans’ theorem, have been actively performed.

LC-DFT calculates exchange energy with the operator 1/*r*_12_ divided into DFT and HF parts as follows:

(1) 1r12=1−erf(μr12)r12+erf(μr12)r12 DFT HF

The first term [left term of Equation (1)] is the inter-electronic short-range Coulomb potential operator that is used for DFT exchange inclusion, and the second term [right term of Equation (1)] is the inter-electronic long-range Coulomb potential operator used for the HF exchange integral. By using them, LC-DFT showed successful improvement of several chemical properties that conventional DFT functionals failed to reproduce [6,10,11,12,13,14,15,16,17,18,19,20,21,22,23,24,25,26,27]. Furthermore, LC-DFT has been shown to successfully reproduce CT excitation energy and its oscillator strength [28,29,30]. In addition, LC-DFT reproduces correct far-nucleus asymptotic behavior, −1/*R*, of the intermolecular CT excitation energies of donor and acceptor with the distance between donor and acceptor [28].

In a previous report [31], we studied the dependences of intramolecular CT excitation energies between-NH_2_ and-NO_2_ and its equidistant intermolecular CT excitation energies between NH_3_ and HNO_2_ without a conjugated polyene chain on CT distances. As a result, as the CT distance between electron donor and acceptor increases, intramolecular CT excitation energy decreases, but intermolecular CT excitation energy increases with asymptotic potential (−1/*R*). We found that this is because the orbital energy gap corresponding to intermolecular CT excitation does not change as the distance increases, but the orbital energy gap corresponding to intramolecular CT excitation decreases because the orbitals of donor and acceptor are delocalized through the conjugated carbon chain. Also, we found that LC-DFT with a large *µ* value (about 0.47) calculates both the intra- and intermolecular CT excitation energies closest to EOM-CCSD/cc-pVTZ compared to LC-DFT with smaller *µ* values or other DFT functionals. Moreover, our recent study clarified that intramolecular CT excitations bridged with alkane chain between electron donor and acceptor are identical to those with no bridge or vacuum [32]. Therefore, CT excitation bridged with alkane chain acts similarly to intermolecular CT excitation.

LC-DFT with an optimally tuned (OT) *µ* parameter using Koopmans’ theorem predicts the frontier orbital energies of a specific molecular system with high accuracy, and many related researchers have made great progress in the efficient prediction of CT excitation energy with LC-DFT [1,33,34,35]. Many theoretical studies on predicting the CT excitation energies of various molecular systems with high accuracy have been performed, but studies that attempt to clarify the dependency of intra- and intermolecular CT excitation energies on the electron-withdrawing strength of an electron acceptor or electron-donating strength of an electron donor have not yet been performed. Also, to the best of our knowledge, no study on the optimal *µ* value that can provide the most accurate intra- and intermolecular CT excitation energy of various electron acceptors (or donors) has been reported, even though there are some reports that the OT method of LC-DFT failed in calculating some photophysical properties [36,37,38].

Therefore, in this article, we will analyze the dependence of intra- and intermolecular CT excitation energies on electron-withdrawing (or -donating) strength and will determine whether an optimally tuned *µ* value obtained using Koopmans’ theorem enables LC-DFT to predict intra- and intermolecular CT excitation energies effectively with various electron acceptors.

## 2. Results and Discussion

### 2.1. The Effect of Electron Acceptors on Intra- and Intermolecular CT Excitation Energies

**Figure 1:**
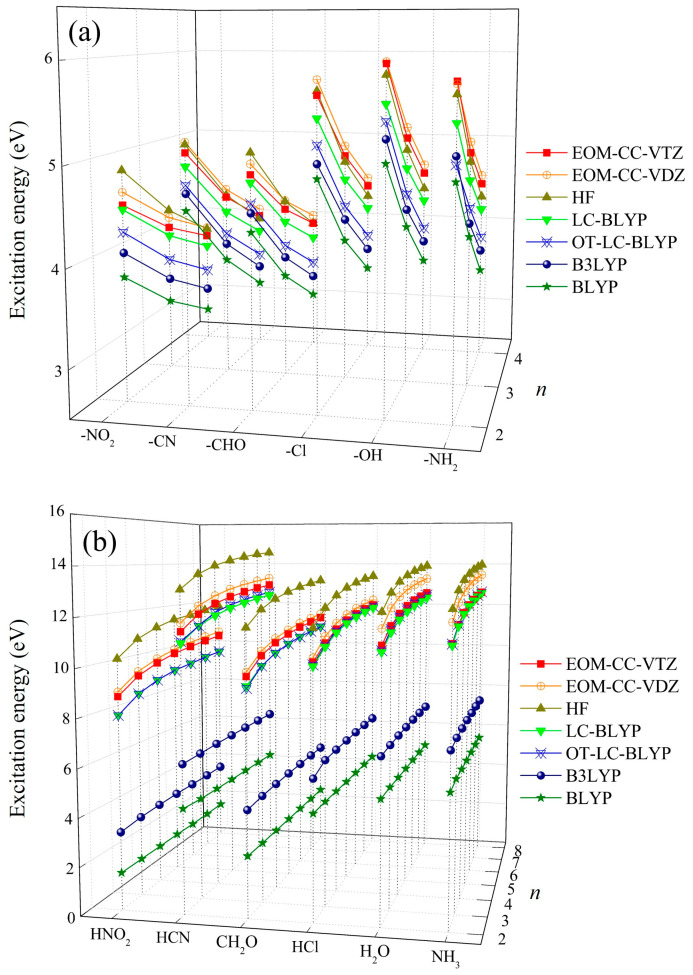
(**a**) Intra- and (**b**) intermolecular CT excitation energies calculated using EOM-CCSD (cc-pVTZ and cc-pVDZ), HF, LC-BLYP, OT-LC-BLYP, B3LYP, and BLYP (cc-pVTZ).

Figure 1 shows the intra- and intermolecular CT excitation energies of H_2_N–(CH=CH)*_n_*–X and H_2_N–H……H–X, respectively, from electron donor, (H)–NH_2_, to various electron acceptors, *X* (NH_2_, OH, Cl, CHO, CN, and NO_2_), with different electron-withdrawing strengths calculated using several quantum chemical methods, such as HF, EOM-CCSD, and various DFT functionals. Both the intra- and intermolecular CT excitation energies calculated using EOM-CCSD/cc-pVTZ are slightly smaller than the EOM-CCSD/cc-pVDZ ones, which indicates that the differences between correlation energies calculated using cc-pVDZ and cc-pVTZ are sufficiently small in both the intra-and intermolecular CT excitation energies.

**Figure 2:**
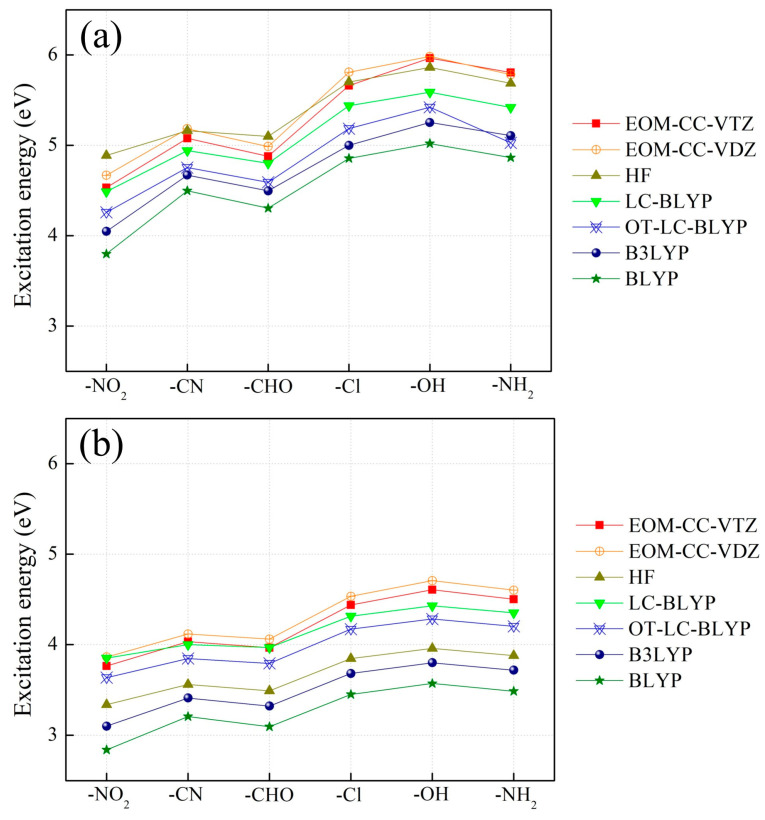
Intramolecular CT excitation energies of H_2_N–(CH=CH)*_n_*–X (X = NH_2_, OH, Cl, CHO, CN, and NO_2_) with (**a**) *n* = 2 and (**b**) *n* = 4 calculated using EOM-CCSD (cc-pVTZ and cc-pVDZ), HF, LC-BLYP, OT-LC-BLYP, B3LYP, and BLYP (cc-pVTZ).

**Figure 3:**
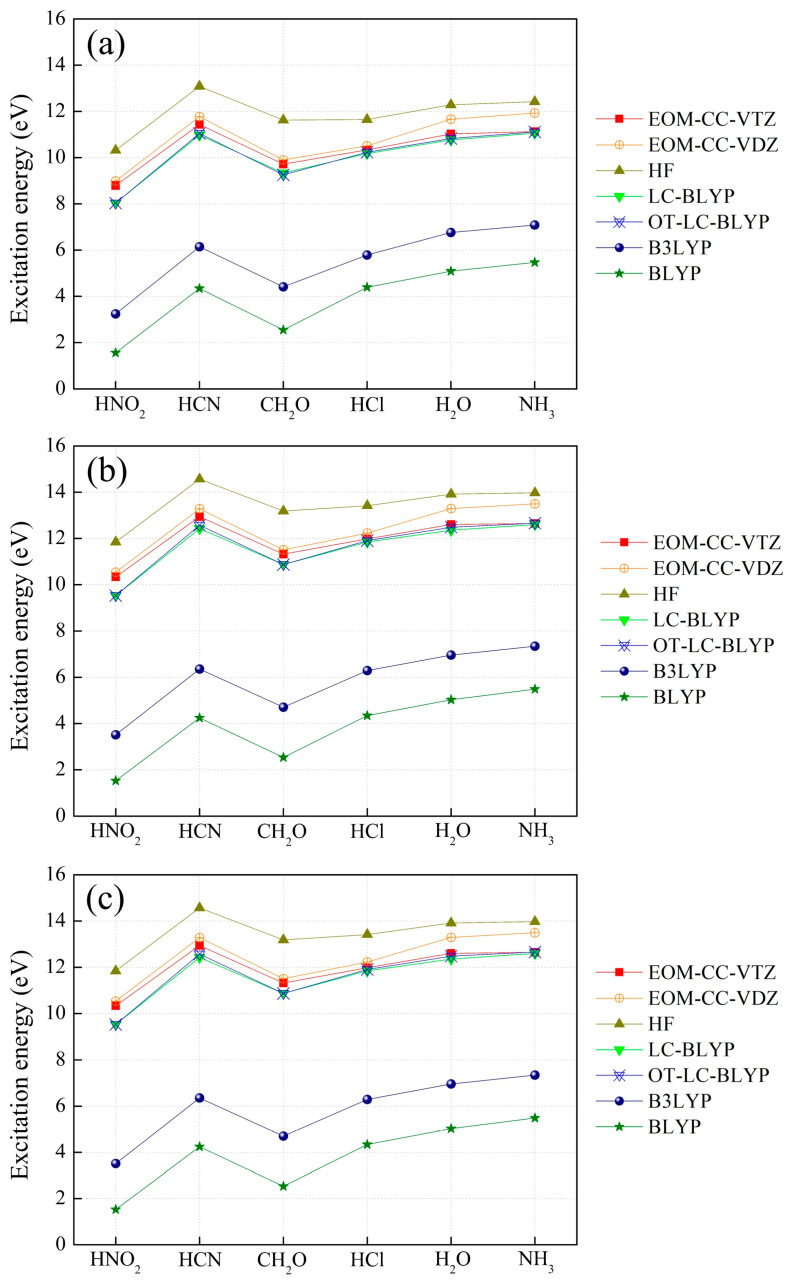
Intermolecular CT excitation energies of H_2_NH……HX (X = NH_2_, OH, Cl, CHO, CN, and NO_2_) with (**a**) *n* = 2, (**b**) *n* = 4, and (**c**) *n* = 8, which are equidistant with H_2_N–(CH=CH)*_n_*–X (X = NH_2_, OH, Cl, CHO, CN, and NO_2_) calculated using EOM-CCSD (cc-pVTZ and cc-pVDZ), HF, LC-BLYP, OT-LC-BLYP, B3LYP, and BLYP (cc-pVTZ).

We examined the effect of the electron-withdrawing strength of *X* on intra- and intermolecular CT excitation energies using the EOM-CCSD/cc-pVTZ ones. As the chain number (*n*) or CT distance (*R*) between electron donor and acceptor increases, the intramolecular CT excitation energies decrease (Figure 2), but the intermolecular CT excitation energies increase regardless of the choice of *X* (Figure 3), as reported in a previous study [31,32]. In order to investigate the effects of electron-withdrawing strength as well as *R* on inter- and intramolecular CT excitation energies, we took as examples for illustrations the inter- and intramolecular CT excitation energies of *n* = 2 and *n* = 4 with *X* = NO_2_, the strongest acceptor, and NH_2_, the weakest acceptor, in Figure 2 and Figure 3. In the case of *n* = 2, the intermolecular CT excitation energy of H_2_NH∙∙∙NH_3_ (11.13 eV) is larger than the H_2_NH∙∙∙HNO_2_ (8.79 eV) by 2.34 eV, and the intramolecular CT excitation energy of H_2_N–(CH=CH)_2_–NH_2_ (5.81 eV) is larger than the H_2_N–(CH=CH)_2_–NO_2_ (4.53 eV) by only 1.27 eV. Similarly, in the case of *n* = 4, the intermolecular CT excitation energy of H_2_NH∙∙∙NH_3_ (12.08 eV) is larger than H_2_NH∙∙∙HNO_2_ (9.75 eV) by 2.33 eV, and the intramolecular CT excitation energy of H_2_N–(CH=CH)_4_–NH_2_ (4.50 eV) is larger than the H_2_N–(CH=CH)_4_–NO_2_ (3.76 eV) by 0.74 eV [Tables S1 and S2]. These results show that as the electron-withdrawing strength of the electron acceptor increases, the intermolecular CT excitation energies significantly decrease (Figure 3). As an exceptional case, the intermolecular CT excitation energies of *X* = CN, which is generally known as a strong electron-withdrawing group, are shown to be similar to the intermolecular CT energies of *X* = NH_2_, which is a strong electron-donating group.

On the other hand, the electron-withdrawing strength of *X* has comparatively little effect on intramolecular CT excitation energies. It is also noticeable that an increase in *R* decreases the effect of *X* on intramolecular CT excitation energies (Figure 2). That is, as the conjugation length of polyene increases, the effect of the polyene conjugation chain on intramolecular CT excitations increases and the effect of the electron-withdrawing strength of *X* diminishes [39]. On the other hand, an increase in *R* does not affect intermolecular CT excitation energies in any cases of *X* (Figure 3).

### 2.2. Assessment of Various DFT Functionals on Intra- and Intermolecular CT Excitation Energy

We assessed DFT functionals for intra- and intermolecular CT excitation energy calculations with the EOM-CCSD/cc-pVTZ ones (Figure 1, Tables S1 and S2). As mentioned in our previous report on the intra- and intermolecular CT excitations of H_2_N–(CH=CH)_2_–NO_2_ and H_2_N–H……H–NO_2_, respectively, the global hybrid DFT (B3LYP) and pure DFT (BLYP) remarkably underestimate both intra- and intermolecular CT excitation energies. On the other hand, LC-DFT [17] (LC-BLYP) calculates both the intra- and intermolecular CT excitation energies significantly close to the EOM-CCSD/cc-pVTZ ones [31]. Unexpectedly, the HF method provides the intermolecular CT excitation ones with larger deviations from the EOM-CCSD ones than the LC-DFT ones, but the intramolecular CT excitation energies are closer to the EOM-CCSD ones than the LC-DFT ones. However, the range of the intramolecular CT excitation energies calculated using all the tested theoretical methods are narrow compared to that of the intermolecular CT energies. Therefore, the deviations in calculated intramolecular CT excitation energies from the EOM-CCSD ones are basically small compared to the intermolecular CT excitation energy calculations.

Then, we examined the performance of the optimal tuning method of the range separation parameter, *µ*, of LC-BLYP using Koopmans’ theorem on inter- and intramolecular CT excitation energies with respect to various *X* and *R*. The optimally tuned *µ* values for both the intra- and intermolecular CT excitation energies with respect to various *X* and *R* are presented in Table 1. The optimal *µ* values for the intermolecular CT excitation systems range from 0.46 to 0.49 and are shown to be independent of a change in *R* as well as *X*. On the other hand, the optimal *µ* values for the intramolecular CT excitation systems are significantly smaller than the default value (0.47) regardless of any choice of *X* (e.g., the optimal *µ* value is 0.18 at *n* = 8 [39,40,41]. Note that the electron-drawing strength of *X* does not influence optimal *µ* values in both the intra- and intermolecular CT excitation systems, which shows that optimal *µ* values are not related to the multitude of orbital energy gaps corresponding to CT excitation as well as electron-withdrawing (or -donating) strength.

**Figure 4:**
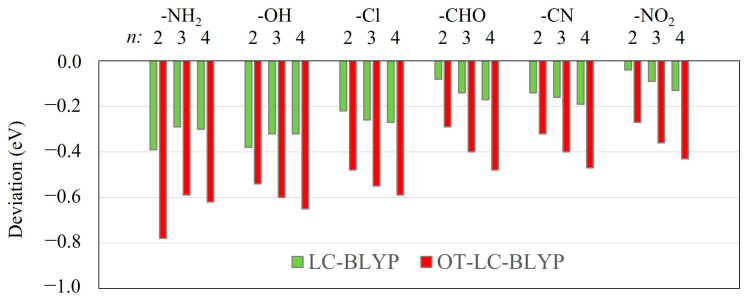
Deviations for intramolecular CT excitation energies of H_2_N−(CH=CH)*_n_*−X (X = NH_2_, OH, Cl, CHO, CN, and NO_2_) with *n* = 2, *n* = 3, and *n* = 4 calculated using LC-BLYP and OT-LC-BLYP (cc-pVTZ) from those calculated using EOM-CCSD/cc-pVTZ.

The intermolecular CT excitation energies calculated using OT-LC-BLYP, whose optimal *µ* values are not different from the default 0.47 value [17], are successfully close to the EOM-CCSD ones irrespective of any choice of *X* and *R* (Figure 1 and Figure 3 and Table S2). However, the intramolecular CT excitation energies calculated using OT-LC-BLYP, whose optimal *µ* values are much smaller than 0.47, are not closer to the EOM-CCSD ones than the LC-BLYP with default *µ* values (*µ* = 0.47) regardless of any choice of *X* and *R* (Figure 1 and Figure 2 and Table S1) even though the differences of the intramolecular CT excitation energies between LC-BLYP and OT-LC-BLYP are not significantly large (by about less than 0.5 eV). Overall, both the intra- and intermolecular CT excitation energies calculated using LC-BLYP with *µ* = 0.47 are closer to the EOM-CCSD/cc-pVTZ ones than OT-LC-BLYP regardless of any choice of *X*. In addition, it is notable that the deviation in intramolecular CT excitation energies obtained using OT-LC-BLYP calculations to EOM-CCSD/cc-pVTZ get larger as *R* increases in Figure 4 and Table S1. In other words, the optimization of the *µ* value of LC-DFT to Koopmans’ theorem is not needed to achieve high-accuracy calculations using LC-DFT on both the intra- and intermolecular CT excitation energy calculations.

### 2.3. Exciton Binding Energy in Intra- and Intermolecular CT Excitation

**Figure 5:**
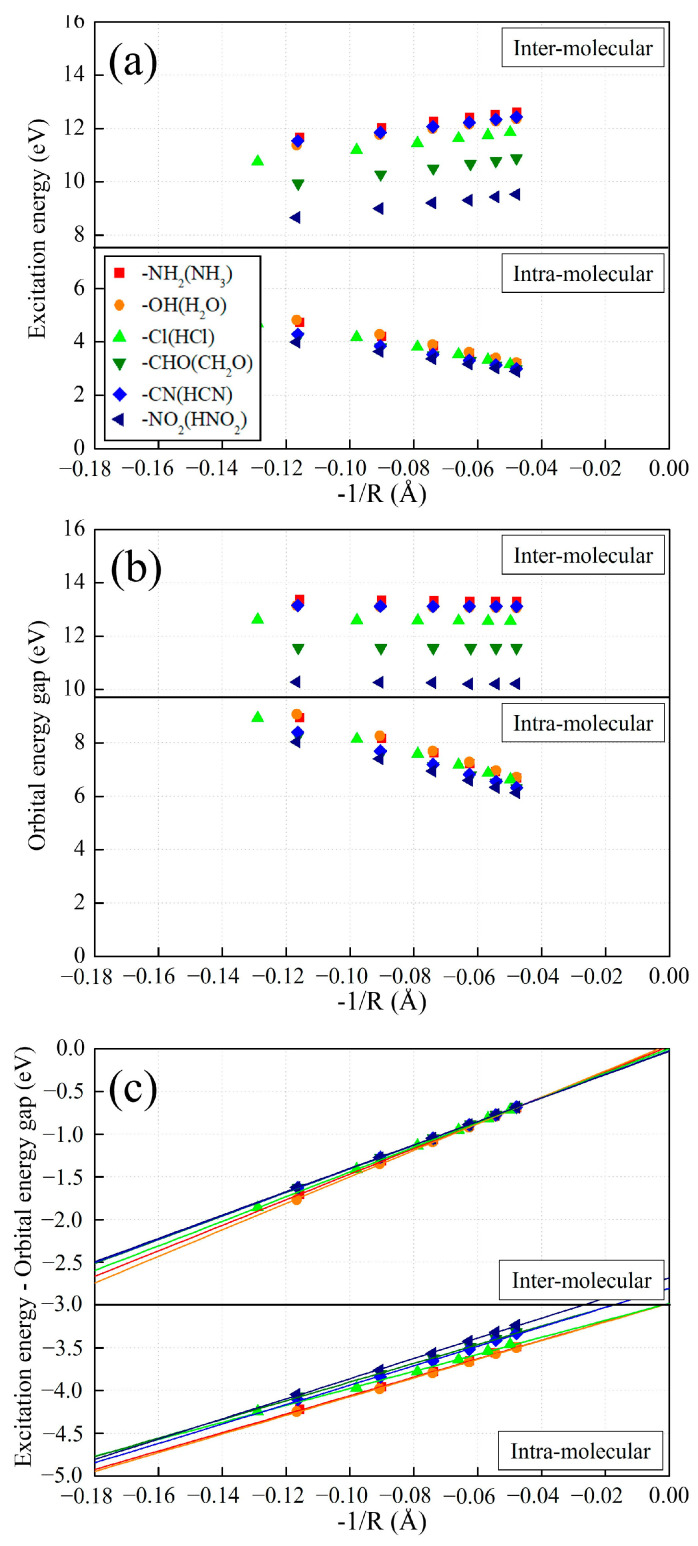
Intra- and intermolecular (**a**) CT excitation energies, (**b**) HOMO-LUMO gaps, and (**c**) exciton binding energies with various electron acceptors plotted against −1/*R* calculated using LC-BLYP. [*n* = 3~8].

We plotted the inter- and intramolecular CT excitation energies from (H–)NH_2_ to various (H–)X calculated using LC-BLYP (Figure 5a), their orbital energy gaps (Figure 5b), and their exciton binding energies (Figure 5c) between orbitals related to the corresponding CT excitation obtained using Equation (10) against −1/*R*. All the plots of the inter- and intramolecular CT excitation energies with respect to various *X* and their energy components are clearly shown to have nearly linear dependences on −1/*R*.

Interestingly, the *y*-intercepts of the intermolecular CT excitation energy plot against −1/*R* are strongly affected by the electron-withdrawing strength of H-*X*. Note that the *y*-intercept of each CT excitation energy plot against −1/*R* means the CT excitation energy converged at *R* → ∞, resulting in the orbital energy gap corresponding to the CT excitation, since excitonic binding energy −1/*R* → 0. Noticeably, the intermolecular CT excitation energies are shifted by the electron-withdrawing strength of H-*X* up to 4–5 eV differences, which is mainly attributed to their orbital energy gaps shifted by the electron-withdrawing strength of H-*X* with the same differences, as shown in Figure 5b.

Meanwhile, the slopes of all the intermolecular orbital energy gaps with respect to various H-*X* against −1/*R* are zero, which indicates that the intermolecular orbital energy gaps are independent of a change in *R* and are not affected by intermolecular CT distance, *R*. The origin of this is that LUMO energies are wholly affected not by *R* but by the H–*X* molecule. In addition, both the slopes of the intermolecular CT excitation energies (Figure 5a) between NH_3_ and H-*X* with respect to various H–*X* against −1/*R* are the same as those of the excitonic binding energies (Figure 5c), which indicates that the intermolecular excitonic binding energy between NH_3_ and H-*X* is the origin of the asymptotic potential of intermolecular CT excitation energy, but the intermolecular orbital energy gaps are not related to the asymptotic potential of intermolecular CT excitation energy. Therefore, it can be concluded that the electron-withdrawing strength of various H-*X* absolutely does not affect the intermolecular exciton binding energy of −1/*R* asymptotic potential, but only the intermolecular orbital energy gap.

Next, we investigated the plots of intramolecular CT excitation energy against −1/*R,* and some conspicuous points compared to the intermolecular CT ones were found. Firstly, the *y*-intercepts of the intramolecular CT excitation energies against −1/*R* are shown to be independent of the electron-withdrawing strength of *X*, contrary to the intermolecular CT excitation ones. That is to say, the electron-withdrawing strength of *X* almost does not affect the intramolecular CT excitation energy when *R* is sufficiently large, as mentioned in the previous section. Secondly, the plots of intramolecular CT excitation energies against −1/*R* with respect to various *X* have the negative slopes, which is solely due to the negative slopes of the intramolecular orbital energy gaps against −1/*R* shown in Figure 5b, while the intramolecular exciton binding energies against −1/*R* have positive slopes. Why do the intramolecular orbital energy gaps have negative slopes against −1/*R*? This is explained in a previous study [31]. That is, it is because the intramolecular orbital energy gaps become narrower as *R* becomes longer, which is originally due to the polyene conjugation chain between electron donor and acceptor that dominates the intramolecular orbital energy gaps more strongly as *R* becomes longer. Thirdly, contrary to the intermolecular CT excitation, the asymptotic lines of intramolecular exitonic binding energies of various *X* against −1/*R* do not converge to zero when −1/*R* goes to zero (*R* goes to infinity), but near to −3.0 as reported in the previous studies in Ref. [31]. The main reason for this seems to be that the CT distance, *R*, between HOMO and LUMO of intramolecular CT excitation in the conjugated chain system becomes shorter than the intermolecular CT distance as the chain number increases because delocalized orbital centers come closer.

### 2.4. Ineffectiveness of Optimal Tuning Method in Intramolecular CT Excitation Calculations

**Figure 6:**
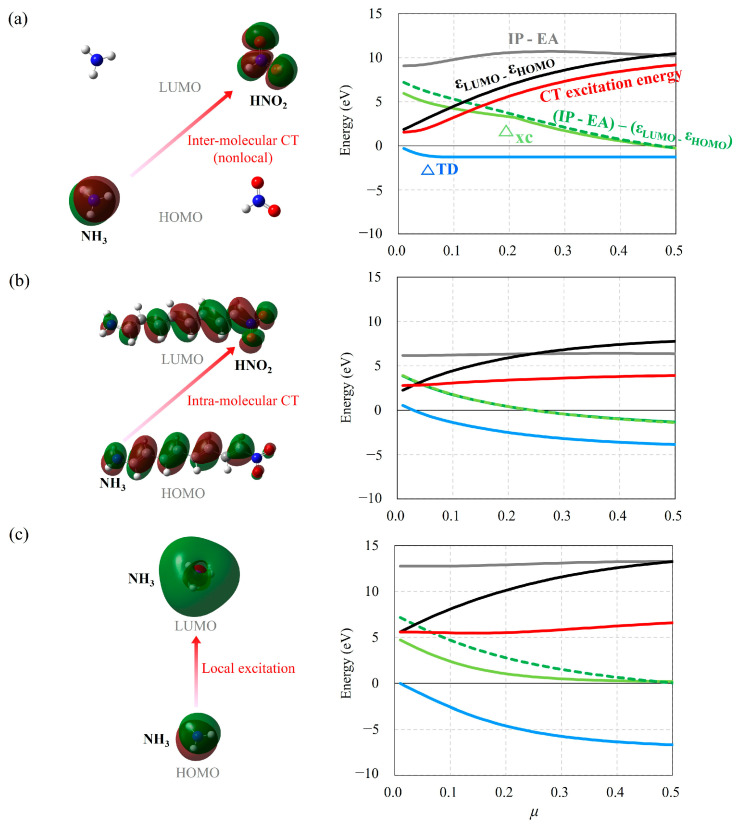
TD-DFT energies, orbital energy gaps, and exciton binding energies (in eV) for (**a**) intermolecular CT excitation from NH_3_ to HNO_2_ of NH_3_……HNO_2_ (*n* = 4), (**b**) intramolecular CT excitation from HOMO to LUMO of H_2_N–(CH=CH)*_n_*–X (*n* = 4), and (**c**) local excitation from HOMO to LUMO of NH_3_ molecule with regard to a change in *µ*.

To find the reason why the optimal tuning method of LC-DFT with Koopman’s theorem is not effective in predicting intramolecular CT excitation energy, unlike the case of intermolecular CT excitation energy [2,3], we investigate the effect of a change in *µ* on inter- and intramolecular CT excitation energies, their orbital energy gaps, and their excitonic binding energies. We scanned the *µ* value for TD-LC-BLYP calculations in intermolecular CT excitation energy of NH_3_……HNO_2_ (*n* = 4), intramolecular CT excitation energy of H_2_N–(CH=CH)*_n_*–NO_2_ (*n* = 4), and local excitation energy of NH_3_ for an illustration (Figure 6 and Table S7).

The evolutions of the intermolecular CT excitation energies and their orbital energy gaps as a function of *µ* are shown to have similar curve shapes with a small difference in Figure 6a. We already know that the reason for this is that intermolecular CT excitation energy is strongly affected by the orbital energy gap, which is increased by an increase in *µ*. To our surprise, the intermolecular exciton binding energy obtained using TD-LC-DFT as Equation (10) is about −1.0 eV regardless of a change in *µ*, which indicates that the intermolecular excitonic binding energy is not affected by the choice of *µ*. Only when *µ* becomes less than 0.05 does the intermolecular excitonic binding energy go to 0.

However, a change in *µ* seems to have little effect on intramolecular CT excitation energy, as shown in Figure 6b. Actually, the evolution of the intramolecular orbital energy gaps as a function of *µ* is quite similar to that of intermolecular ones. However, while the intermolecular exciton binding energy is independent of a change in *µ*, the intramolecular exciton binding energy is largely dependent on a change in *µ*, which eventually is the main reason why intramolecular CT excitation energy is not substantially affected by a change in *µ*. Therefore, the different dependence of the intramolecular CT excitation energy from the intermolecular one on a change in *µ* seems to be mainly due to the different dependence of their exciton binding energy on a change in *µ*.

It is noticeable that this behavior seen in intramolecular CT excitation is also observed in local excitation. Figure 6c shows how local excitation energy behaves as a function of *µ* in a manner similar to intramolecular CT excitation energy, which indicates that intramolecular CT excitation is fundamentally similar to local excitation. The electronic transition shown in the Figure 6c takes place from the HOMO of NH_3_ to the LUMO. As mentioned earlier, the evolutions of local excitation energy, its following orbital energy gap, and exciton binding energy as a function of a change in *µ* have behaviors analogous to those of intramolecular CT excitation ones. In other words, as *µ* increases, the orbital energy gap increases, exciton binding energy decreases, and eventually local excitation energy has little evolution compared to intramolecular CT excitation energy.

Here, it should be mentioned that Ref. [3] explains that the origin of the increases in intermolecular CT excitation energy with respect to an increase in *µ* is asymptotic behavior with respect to distance (*R*) between donor, NH_3_, and acceptor, HNO_2_, which is evidently due to exciton binding energy with −1/*R* behavior. In detail, it is written in Ref. [3] that “*The reason why LE (local excited) excitations and CT excitations show so markedly different evolutions with respect to ω (µ) is related to the fact that the CT states are very sensitive to the nature of the asymptotic potential, which is strongly affected by the choice of ω (µ), whereas the LE states probe the asymptotic potential to a much smaller degree*”. However, we inevitably should indicate here that the statement “*the asymptotic potential is strongly affected by the choice of ω (µ), whereas the LE states probe is not so much*” is mistaken. On the contrary, as clearly observed in Figure 6a, the intermolecular exciton binding energy obtained using TD-DFT calculations does not depend on a change in *µ* and is invariable as far as the *µ* value is not less than 0.1. Only *R* can affect the amount of exciton binding energy between electron donor and acceptor. The intramolecular exciton binding energy decreases with an increase in *µ*, as shown in Figure 6b. Therefore, the statement in Ref. [3] should be corrected as “*The reason why LE (local excited) excitations and CT excitations show so markedly different evolutions with respect to ω (µ) is related to the fact that the CT states probe the asymptotic potential (or exciton binding energy) to a much smaller degree, whereas the LE states are very sensitive to the nature of the asymptotic potential (or exciton binding energy), which is strongly affected by the choice of ω (µ)*”.

Why do the excitonic binding energy contributions in the local excitation energies as well as the intramolecular CT excitation by TD-LC-DFT through Equation (10) become larger (or more negative) with respect to an increase in *µ* in Figure 6b? In local excitations, the electron transition takes place from the HOMO of NH_3_ to the LUMO. In other words, one electron is removed from HOMO, and the electron is added to LUMO in the electron transition, which elevates HOMO energy and lowers LUMO energy. We call this energy effect caused by electron transition from one orbital to the other orbital the “orbital relaxation effect”. In the case of intramolecular CT excitation as well as local excitation, HF, hybrid-DFT, and LC-DFT include this orbital relaxation effect by TD calculations more as the HF exchange integral included in the exchange functional decreases. As a result, optical band gaps of local and intramolecular CT excitations obtained using TD calculations of HF, hybrid-DFT, and LC-DFT become similar.

Optimal fitting of the *µ* parameter of LC-DFT to Koopmans’ theorem allows TD-LC-DFT to calculate intermolecular CT excitation energy with relatively high accuracy because it calculates corresponding frontier orbital energies with their orbital relaxation effects included correctly. The *IP*-*EA* values obtained from the energies of cation (A^+^), anion (A^−^), and neutral molecule (A) using ΔSCF with *µ* parameter scanned show that they become identical to ε_LUMO_–ε_HOMO_ at *µ* = 0.5 (Figure 6a), which means that LC-DFT with that value of *µ* provides the orbital energy gap with the orbital relaxation effect included correctly. Moreover, since TD-LC-DFT can calculate −1/*R* asymptotic potential between electron donor and acceptor correctly, the intermolecular CT excitation energies can be calculated with high accuracy. Interestingly, the evolutions of Δ_XC_ values obtained using Equation (25) as a function of *µ* are very similar to those of *IP*–*EA*–(ε_LUMO_–ε_HOMO_), which indicates that the minimization of the Δ_XC_ value with changing *µ* corresponds to making orbital energy include orbital relaxation correctly.

Meanwhile, both the *IP*–*EA*–(*ε*_LUMO_–*ε*_HOMO_) and Δ_XC_ values of the intramolecular CT excitation energies become zero at about *µ* = 0.25 even though it is shown that TD-LC-DFT with a larger *µ* value provides similar results with the EOM-CCSD ones in Section 2.2. In the case of the local excitation of NH_3_, both the *IP*–*EA*–(ε_LUMO_–ε_HOMO_) and Δ_XC_ values simultaneously become zero at large *µ* value (0.45). The charges of cation and anion of the conjugated chain systems in the intramolecular CT excitation are expected to be delocalized, which reduces energy changes in the cation and anion caused by the charge, which will make the *µ* value optimized less than those obtained in the intermolecular CT excitation in the conjugated system or local excitations in a small molecule [38,42]. Therefore, to overcome this ineffectiveness of optimal tuning methods of LC-DFT in predicting intramolecular CT excitation energy, we should solve this delocalization problem in conjugated molecules [43].

## 3. Theory

### 3.1. Time-Dependent Density Functional Theory with Hybrid- and LC-DFTs

In TD-DFT calculations, excitation energy ω and the corresponding excitation vectors **X** and **Y** are usually obtained by solving a non-Hermitian eigenvalue equation [9,44,45].

(2) $$\left(\begin{array}{cc} A & B\\ B & A \end{array}\right)\left(\begin{array}{c} X\\ Y \end{array}\right)=\omega \left(\begin{array}{cc} 1 & 0\\ 0 & -1 \end{array}\right)\left(\begin{array}{c} X\\ Y \end{array}\right)$$

The elements of the matrices **A** and **B** are

(3) $$A_{ia\sigma ,jb\tau }=\delta_{ij}\delta_{ab}\delta_{\sigma \tau }(\varepsilon_{a\sigma }-\varepsilon_{i\sigma })+K_{ia\sigma ,jb\tau }$$

where $\varepsilon_{i\sigma }$ is the *i*th Kohn–Sham *σ*-spin orbital energy, and

(4) $$B_{ia\sigma ,jb\tau }=K_{ia\sigma ,bj\tau }$$

where matrix element $K_{ia\sigma ,jb\tau }$ in Equations (3) and (4) is given by

(5) $$K_{ia\sigma ,jb\tau }=(ia\sigma |bj\tau )+\int \int \psi_{i\sigma }^{*}\left(r_{1}\right)\psi_{a\sigma }^{*}\left(r_{2}\right)\frac{\delta^{2}E_{xc}}{\delta \rho_{\sigma }\left(r_{1}\right)\delta \rho_{\tau }\left(r_{2}\right)}\psi_{j\tau }\left(r_{1}\right)\psi_{b\tau }\left(r_{2}\right)d^{3}r_{1}d^{3}r_{2}-K_{ia\sigma ,jb\tau }^{\mathrm{HF}}$$

As usual, indices *i*, *j*, and *a*, *b*, label occupied and virtual orbitals, respectively. In Equation (5), the first term on the right side is a two-electron repulsion integral,

(6) $$(ia\sigma |bj\tau )=\int \int \psi_{i\sigma }^{*}\left(r_{1}\right)\psi_{a\sigma }\left(r_{1}\right)\frac{1}{r_{12}}\psi_{b\tau }^{*}\left(r_{2}\right)\psi_{j\tau }\left(r_{2}\right)d^{3}r_{1}d^{3}r_{2}$$

and $K_{ia\sigma ,jb\tau }^{\mathrm{HF}}$ is the mixed HF exchange integral term; for pure functionals, $K_{ia\sigma ,jb\tau }^{\mathrm{HF}}$ = 0, and for conventional hybrid functionals,

(7) $$K_{ia\sigma ,jb\tau }^{\mathrm{HF}}=c_{x}\delta_{\sigma \tau }\int \int \psi_{i\sigma }^{*}\left(r_{1}\right)\psi_{a\sigma }^{*}\left(r_{2}\right)\frac{1}{r_{12}}\psi_{j\tau }\left(r_{1}\right)\psi_{b\tau }\left(r_{2}\right)d^{3}r_{1}d^{3}r_{2}$$

where *c_x_* is a constant mixing rate of HF exchange integral in global hybrid functionals, such as B3LYP and PBE0. TD-LC-DFT includes a nonzero $K_{ia\sigma ,jb\tau }^{\mathrm{HF}}$, due to the HF exchange terms in Equation (1),

(8) $$K_{ia\sigma ,jb\tau }^{\mathrm{HF}}=\delta_{\sigma \tau }\int \int \psi_{i\sigma }^{*}\left(r_{1}\right)\psi_{a\sigma }^{*}\left(r_{2}\right)\left[\frac{\mathrm{erf}\left(\mu r_{12}\right)}{r_{12}}\right]\psi_{j\tau }\left(r_{1}\right)\psi_{b\tau }\left(r_{2}\right)d^{3}r_{1}d^{3}r_{2}$$

### 3.2. Asymptotic Potential and Excitonic Bindng Energy

From Equations (2)–(8), we can approximate the optical CT excitation gap between occupied (O_MO_) and unoccupied molecular orbitals (U_MO_) (e.g.*,* O_MO_ = HOMO and U_MO_ = LUMO) [31,32,46] as

*ω*_CT_^TD^ ≈ *ε*_U_ − *ε*_O_ + (O_MO_U_MO_|1/*r*_12_|O_MO_U_MO_) − (O_MO_O_MO_|*O*(*r*_12_)|U_MO_U_MO_) (9)

Here, the third term on the right side originates from the two-electron repulsion integral term shown in Equation (6), and the fourth term on the right side originates from the HF exchange interaction shown in Equations (7) and (8). *O*(*r*_12_) is a two-electron HF exchange operator. For example, *O*(*r*_12_) of B3LYP is *c_x_*/*r*_12_ (*c_x_* = 0.2), that of LC-DFT is erf(*μr*_12_)/*r*_12_, and that of the pure functional is 0. The excitonic binding energy (*E*^exciton^) [46,47] between O_MO_ and U_MO_ calculated in TD-DFT can be considered as

*E*^exciton^ ≈ *ω*_CT_^TD^ − (*ε*_U_–*ε*_O_) (10)

In the intermolecular CT excitation and intramolecular CT excitation of polyalkane,

*E*^exciton^ ≈ −(O_MO_O_MO_|*O*(*r*_12_)|U_MO_U_MO_) (11)

because (O_MO_U_MO_|1/*r*_12_|O_MO_U_MO_) will disappear quickly as the distance between donor and acceptor (*R*) increases, in that the overlap between HOMO and LUMO is too small. Moreover, the excitonic binding energies of pure and screened hybrid-DFT functionals become zero, in that (O_MO_O_MO_|*O*(*r*_12_)|U_MO_U_MO_) also disappear quickly. In the case of LC-DFT, (O_MO_O_MO_|*O*(*r*_12_)|U_MO_U_MO_) becomes −1/*R*. Therefore, in the case of intermolecular CT excitations, the excitonic binding energy of LC functionals will be like

*E*^exciton^ ≈ −1/*R* (12)

which is known as an asymptotic potential of CT excitation energy.

### 3.3. Derivative Discontinuity of Approximated Exchange–Correlation Functionals

In previous studies for a system with a fractional electron number [48,49,50], it was shown that the derivative of the total energy with respect to electron number is the chemical potential and its discontinuous derivatives at an integer number of an electron number on both sides gives the following relations, which is also related to conceptual DFT:

(13) $${\left(\frac{\partial E}{\partial N}\right)}_{+}=\underset{f\to 0^{+}}{\lim}\frac{dE(N+f)}{df}=E(N+1)-E(N)=-EA$$

and

(14) $${\left(\frac{\partial E}{\partial N}\right)}_{-}=\underset{f\to 0^{-}}{\lim}\frac{dE(N+f)}{df}=E(N)-E(N-1)=-IP$$

Additionally, Refs. [51,52,53,54,55] show that in exact DFT

(15) $$\varepsilon_{\mathrm{HOMO}}=-IP$$

but in the case of the approximated exchange–correlation functional and optimized effective potential (OEP) orbitals

(16) $$\varepsilon_{\mathrm{LUMO}}=-EA-\langle \mathrm{LUMO}\left|\sum_{\mathrm{XC}}\left(\left\{\varepsilon_{\mathrm{LUMO}}\right\}\right)-v_{\mathrm{XC}}\right|\mathrm{LUMO}\rangle$$

where Σ_XC_ is an energy-dependent exchange–correlation potential or exchange–correlation self-energy operator that includes correlation and relaxation effects, self-interaction correction, and so on, and *v*_XC_ is the approximated exchange–correlation potential, and in other references it is expressed as

(17) $$\varepsilon_{\mathrm{LUMO}}=-EA-\langle \mathrm{LUMO}\left|v_{\mathrm{XC}}^{\mathrm{nonlocal}}\left(r,r^{\prime }\right)-v_{\mathrm{XC}}\left(r\right)\right|\mathrm{LUMO}\rangle$$

where it is the nonlocal exchange–correlation potential [55]. Actually, the second terms on the right side of Equations (16) and (17) are different expressions of the approximated exchange–correlation functional derivative discontinuity [50,54,55,56,57],

(18) $$\Delta_{\mathrm{XC}}=v_{XC}^{\left(+\right)}\left(r\right)-v_{XC}^{\left(-\right)}\left(r\right)$$

where

(19) $$v_{XC}^{\left(\pm \right)}\left(r\right)={\underset{f\to 0^{\pm }}{\lim}\frac{\delta E_{XC}\left[\rho \right]}{\delta \rho \left(r\right)}|}_{N\pm \delta }$$

Meanwhile, according to Equations (4) and (6), the HOMO energy of the anion, *A*^−^, which is an *N* + 1-electron system, is –*EA* as

(20) $$\varepsilon_{\mathrm{HOMO}}(A^{-}:N+1)=-EA$$

if the derivative linearity of the total energy with respect to the fractional electron number is maintained [58]. However, in the case of the neutral molecule, *A*, which is an *N*-electron system, LUMO energy is not –*EA* as

(21) $$\varepsilon_{\mathrm{HOMO}}(A^{-}:N+1)=\varepsilon_{\mathrm{LUMO}}(A:N)+\Delta_{XC}$$

Note that the addition of an electron to the LUMO of the *N*-electron system makes the HOMO of the *N* + 1-electron system, and the related energy differences are expressed in the second terms on the right side of Equations (16) and (17).

Therefore,

(22) $$\varepsilon_{\mathrm{LUMO}}(A:N)+\Delta_{XC}=-EA$$

However, if the approximated exchange–correlation functional and its kernel are exact, as shown in Equation (11) of Ref [59], the ∆_XC_ will become zero on the grounds of Janak’s theorem and the derivative linearity of the total energy [6]. Therefore, in exact DFT

(23) $$\varepsilon_{\mathrm{LUMO}}=-EA$$

Therefore,

(24) $$\varepsilon_{\mathrm{HOMO}}(A^{-}:N+1)=\varepsilon_{\mathrm{LUMO}}(A:N)+\Delta_{XC}$$

and

(25) $$\Delta_{XC}=\varepsilon_{\mathrm{HOMO}}(A^{-}:N+1)-\varepsilon_{\mathrm{LUMO}}(A:N)$$

Equation (25) provides one of the methods we can use to assess the approximated exchange–correlation functional on calculating frontier orbital energies.

## 4. Computational Details

We obtained H_2_N–(CH=CH)*_n_*–*X* (*n* = 2~8) polyene geometries with various electron-withdrawing groups (*X* = NH_2_, OH, Cl, CHO, CN, and NO_2_) optimized using B3LYP/aug-cc-pVTZ [60] for intra-molecular CT excitation calculations. Then, we obtained H_2_NH…HX structures by removing the polyene conjugation backbones of H_2_N–(CH=CH)*_n_*–X (*n* = 2~8) followed by adding an H atom to –NH_2_ and –X [31,32]. The lengths between the central atom and H were set up with 1.01 Å (–NH_2_), 1.00 Å (–OH), 1.28 Å (–Cl), 1.10 Å (–CHO), 1.07 Å (–CN), and 1.01 Å (–NO_2_), respectively, which are the average bond lengths between other H atoms and the central atoms of each molecule. The CT distances (*R*) between electron donors and acceptors were estimated from the central atoms of the electron donor to those of the acceptor. However, since the central orbital of HCl is closer to the H atom than other electron acceptor molecules, we set the distance between N in NH_3_ and H in HCl as *R*.

We obtained CT excitation energies using TD-DFT calculations with various functionals (BLYP, B3LYP, and LC-BLYP) as well as wavefunction theory (TD-HF and EOM-CCSD). Also, optimally tuned (OT) [33] LC-BLYP calculations where *µ* is optimized with Koopmans’ theorem were also performed.

Most of the intramolecular CT excitations correspond to the electronic transitions from HOMO to LUMO except for some CT excitation cases calculated using EOM-CCSD, HF, and BLYP when *X* = NH_2_, OH, CHO with *n* = 2. However, the intermolecular CT excitations do not correspond to the electronic transition from HOMO to LUMO in some cases. We investigated the molecular orbital shapes of related electron transitions to confirm the intermolecular CT excitations from the molecular orbital of electron donor to that of the acceptor. The numbers of the CT orbitals and their shapes are shown in Tables S8 and S9 and Figures S8–S17.

All the DFT and HF calculations were performed using cc-pVTZ, but EOM-CCSD was calculated using cc-pVDZ and cc-pVTZ. All calculations were executed on the official version of the Gaussian16 program [61].

## 5. Conclusions

In this study, we analyzed inter- and intramolecular CT excitation energies, their orbital energy gaps, and their exciton binding energies with changing electron acceptors that have various electron-withdrawing strengths and conjugated carbon chain lengths or CT distances. Both inter- and intramolecular CT excitation energies show decreased CT excitation energy with regard to an increase in electron-withdrawing strength. However, in contrast to intermolecular CT excitation energy, the effect of electron-withdrawing strength on intramolecular CT excitation energy is very small because intramolecular CT has low dependence on electron-withdrawing strength.

Also, we confirmed that the orbital energy gap between electron donor and acceptor orbitals play a significant role in determining intermolecular CT excitation energy, in that asymptotic potential of −1/*R* calculated using TD-DFT, which is also called excitonic binding energy, is not dependent on the range separation parameter value. Therefore, the optimal tuning method of the range separation parameter is significantly effective for predicting intermolecular CT excitation energy. However, accurate calculations of anion and cation energies are difficult in the intramolecular CT excitation of conjugated chain systems since the delocalization of charge and its reduced charge effect on the total energy of cation and anion disturb computations of *IP* and *EA* in the optimal tuning method. Therefore, the ineffectiveness of optimal tuning methods of LC-DFT in predicting intramolecular CT excitation energy can be overcome by an accurate delocalization error in conjugated molecules. Moreover, it is recommended to use a larger *µ* value rather than optimally tuned *µ* values to calculate high-accuracy intramolecular CT excitation energies with a polyene conjugation chain.

## Figures and Tables

**Table 1:** Optimally tuned *µ* values for intra- and intermolecular CT excitations with various electron acceptors using Koopman’s theorem.

*n*	-X(HX)	Intra	Inter	*n*	-X(HX)	Intra	Inter
2	-NH_2_(NH_3_)	0.29	0.48	6	-NH_2_(NH_3_)	0.21	0.48
	-OH(H_2_O)	0.30	0.48		-OH(H_2_O)	0.21	0.49
	-Cl(HCl)	0.29	0.48		-Cl(HCl)	0.20	0.48
	-CHO(CH_2_O)	0.29	0.46		-CHO(CH_2_O)	0.20	0.47
	-CN(HCN)	0.29	0.48		-CN(HCN)	0.20	0.49
	-NO_2_(HNO_2_)	0.30	0.47		-NO_2_(HNO_2_)	0.20	0.47
3	-NH_2_(NH_3_)	0.26	0.48	7	-NH_2_(NH_3_)	0.19	0.48
	-OH(H_2_O)	0.27	0.49		-OH(H_2_O)	0.20	0.49
	-Cl(HCl)	0.26	0.48		-Cl(HCl)	0.19	0.48
	-CHO(CH_2_O)	0.26	0.47		-CHO(CH_2_O)	0.19	0.47
	-CN(HCN)	0.26	0.47		-CN(HCN)	0.19	0.49
	-NO_2_(HNO_2_)	0.26	0.47		-NO_2_(HNO_2_)	0.19	0.47
4	-NH_2_(NH_3_)	0.24	0.48	8	-NH_2_(NH_3_)	0.18	0.48
	-OH(H_2_O)	0.24	0.49		-OH(H_2_O)	0.18	0.49
	-Cl(HCl)	0.24	0.48		-Cl(HCl)	0.18	0.48
	-CHO(CH_2_O)	0.24	0.47		-CHO(CH_2_O)	0.18	0.47
	-CN(HCN)	0.24	0.49		-CN(HCN)	0.18	0.49
	-NO_2_(HNO_2_)	0.24	0.47		-NO_2_(HNO_2_)	0.18	0.47
5	-NH_2_(NH_3_)	0.22	0.48				
	-OH(H_2_O)	0.22	0.49				
	-Cl(HCl)	0.22	0.48				
	-CHO(CH_2_O)	0.22	0.47				
	-CN(HCN)	0.22	0.49				
	-NO_2_(HNO_2_)	0.22	0.47				

## Data Availability

The original contributions presented in the study are included in the article/Supplementary Materials, further inquiries can be directed to the corresponding author.

## Supplementary Materials

The following supporting information can be downloaded at: https://www.mdpi.com/article/10.3390/molecules29184423/s1, **Table S1.** Intramolecular CT excitation energy between electron-donating orbital corresponding to -NH_2_ and electron-withdrawing orbitals corresponding to various electron acceptors (eV). Values in parentheses are errors of CT excitation energies from EOM-CCSD. **Table S2.** Intermolecular CT excitation energy from electron-donating orbital corresponding to NH_3_ to electron-withdrawing orbitals corresponding to various electron acceptors (eV). Values in parentheses are errors of CT excitation energies from EOM-CCSD. **Table S3.** Intramolecular orbital energy gaps between electron-donating orbital corresponding to -NH_2_ and electron-withdrawing orbitals corresponding to various electron acceptors (eV). **Table S4.** Intermolecular orbital energy gaps between electron-donating orbital corresponding to NH_3_ and electron-withdrawing orbitals corresponding to various electron acceptors (eV). **Table S5.** Intramolecular exciton binding energies between electron-donating orbital corresponding to -NH_2_ and electron-withdrawing orbitals corresponding to various electron acceptors (eV). **Table S6.** Intermolecular exciton binding energies between electron-donating orbital corresponding to NH_3_ and electron-withdrawing orbitals corresponding to various electron acceptors (eV). **Table S7.** Orbital energy gap, CT excitation energy and exciton binding energy (eV) of intra- and intermolecular CT excitations from -NH_2_ (NH_3_) to -NO_2_ (HNO2) with *n* = 4 in a change in *μ* value. **Table S8.** Molecular orbitals corresponding to intramolecular CT excitations from -NH_2_ and various electron acceptor. (H is HOMO and L is LUMO). **Table S9.** Molecular orbitals corresponding to intramolecular CT excitations from NH_3_ and various electron acceptor. (H is HOMO and L is LUMO). **Figure S1.** Orbital energy gaps corresponding to (a) intramolecular and (b) intermolecular CT excitations between electron-donating and electron-withdrawing orbitals calculated using HF, LC-BLYP, OT-LC-BLYP, B3LYP and BLYP. **Figure S2.** Intramolecular CT excitation energies from electron-donating to electron-withdrawing orbitals with (a) *n* = 2, (b) *n* = 3, and (c) *n* = 4 calculated using EOM-CCSD, HF, LC-BLYP, OT-LC-BLYP, B3LYP and BLYP. **Figure S3.** Intermolecular CT excitation energies from electron-donating to electron-withdrawing orbitals with (a) *n* = 2, (b) *n* = 3, (c) *n* = 4, (d) *n* = 5, (e) *n* = 6, (f) *n* = 7, and (g) *n* = 8 calculated using EOM-CCSD, HF, LC- BLYP, OT-LC-BLYP, B3LYP and BLYP. **Figure S4.** Orbital energy gaps between electron-donating and electron-withdrawing orbitals in intramolecular CT excitations with (a) *n* = 2, (b) *n* = 3, and (c) *n* = 4 calculated using HF, LC-BLYP, OT-LC- BLYP, B3LYP and BLYP. **Figure S5.** Orbital energy gaps between electron-donating and electron-withdrawing orbitals of intermolecular CT excitations with (a) *n* = 2, (b) *n* = 3, (c) *n* = 4, (d) *n* = 5, (e) *n* = 6, (f) *n* = 7, and (g) *n* = 8 calculated using HF, LC-BLYP, OT-LC-BLYP, B3LYP and BLYP. **Figure S6.** Intramolecular HOMO and LUMO energies of (a) H_2_N–(CH=CH)*_n_*–NH_2_ and (b) H2N–(CH=CH)*_n_*–NO_2_ and intermolecular HOMO and LUMO energies of (c) H_2_N–H……H–NH2 and (d) H_2_N–H……H–NO_2_ with regard to *n* calculated using LC-BLYP. The HOMO and LUMO energies of NH_3_ molecule in (a) and (c) and the HOMO energy of NH_3_ and the LUMO of HNO_2_ in (b) and (d) are displayed in both sides. **Figure S7.** (a) HOMO and LUMO energies of electron acceptors as single molecule of (HX) (b) HOMO and LUMO energies of their combination with NH_3_ molecule for intermolecular CT excitations [H_2_N–H……H–X] (*n* = 4), (c) HOMO and LUMO energies for intramolecular CT excitations [H_2_N–(CH=CH)*_n_*–NO_2_] (*n* = 4). **Figure S8.** MO shapes of intramolecular CT excitations from -NH_2_ to various electron acceptors with *n* = 2 calculated using HF. **Figure S9.** MO shapes of intramolecular CT excitations from -NH_2_ to various electron acceptors with *n* = 3 calculated using HF. **Figure S10.** MO shapes of intramolecular CT excitations from -NH_2_ to various electron acceptors with *n* = 4 calculated using HF. **Figure S11.** MO shapes of intermolecular CT excitations from NH_3_ to various electron acceptors with *n* = 2 calculated using HF. **Figure S12.** MO shapes of intermolecular CT excitations from NH_3_ to various electron acceptors with *n* = 3 calculated using HF. **Figure S13.** MO shapes of intermolecular CT excitations from NH_3_ to various electron acceptors with *n* = 4 calculated using HF. **Figure S14.** MO shapes of intermolecular CT excitations from NH_3_ to various electron acceptors with *n* = 5 calculated using HF. **Figure S15.** MO shapes of intermolecular CT excitations from NH_3_ to various electron acceptors with *n* = 6 calculated using HF. **Figure S16.** MO shapes of intermolecular CT excitations from NH_3_ to various electron acceptors with *n* = 7 calculated using HF. **Figure S17.** MO shapes of intermolecular CT excitations from NH_3_ to various electron acceptors with *n* = 8 calculated using HF.
