# Supplementary material for: Why Does the Optimal Tuning Method of the Range Separation Parameter of a Long-Range Corrected Density Functional Fail in Intramolecular Charge Transfer Excitation Calculations?
Source: Molecules. 2024 Sep 18;29(18):4423. doi: 10.3390/molecules29184423 (PMC11434625; doi:10.3390/molecules29184423)
Supplement: Supplementary file 1 [file molecules-29-04423-s001.zip › molecules-3190618-supplementary.pdf]

# Supplemental Tables and Figures

*to accompany*

**“Why does the optimal tuning method of the range separation parameter of a long-range corrected density functional fail in intramolecular charge transfer excitation calculations?”**

by

**Han-Seok Bae, Dae-Hwan Ahn, and Jong-Won Song***

*Molecules*

**Table S1.** Intramolecular CT excitation energy between electron-donating orbital corresponding to $-NH_2$ and electron-withdrawing orbitals corresponding to various electron acceptors (eV). Values in parentheses are errors of CT excitation energies from EOM-CCSD.

| *n* | Distance R(Å) | -X | EOM-CCSD (cc-pVTZ) | EOM-CCSD (cc-pVDZ) | LC-BLYP ($\mu$=0.47) | OT-LC-BLYP | HF | B3LYP | BLYP |
|---|---|---|---|---|---|---|---|---|---|
| 2 | 6.19 | $-NH_2$ | 5.81 | 5.78 (-0.02) | 5.42 (-0.39) | 5.03 (-0.78) | 5.69 (-0.12) | 5.11 (-0.70) | 4.86 (-0.94) |
| | 6.12 | -OH | 5.97 | 5.98 (0.02) | 5.59 (-0.38) | 5.42 (-0.54) | 5.86 (-0.10) | 5.25 (-0.71) | 5.02 (-0.95) |
| | 5.29 | -Cl | 5.66 | 5.81 (0.15) | 5.44 (-0.22) | 5.18 (-0.48) | 5.70 (0.04) | 5.00 (-0.66) | 4.86 (-0.80) |
| | 6.16 | -CHO | 4.88 | 4.99 (0.11) | 4.80 (-0.08) | 4.59 (-0.29) | 5.10 (0.22) | 4.50 (-0.38) | 4.30 (-0.58) |
| | 6.14 | -CN | 5.08 | 5.18 (0.11) | 4.94 (-0.14) | 4.76 (-0.32) | 5.16 (0.08) | 4.67 (-0.41) | 4.50 (-0.58) |
| | 6.12 | $-NO_2$ | 4.53 | 4.67 (0.14) | 4.49 (-0.04) | 4.26 (-0.27) | 4.89 (0.36) | 4.05 (-0.49) | 3.80 (-0.73) |
| 3 | 8.65 | $-NH_2$ | 5.00 | 5.12 (0.11) | 4.71 (-0.29) | 4.41 (-0.59) | 4.91 (-0.09) | 4.25 (-0.75) | 4.11 (-0.89) |
| | 8.57 | -OH | 5.14 | 5.25 (0.11) | 4.82 (-0.32) | 4.55 (-0.60) | 5.02 (-0.13) | 4.38 (-0.77) | 4.19 (-0.95) |
| | 7.76 | -Cl | 4.94 | 5.05 (0.11) | 4.68 (-0.26) | 4.55 (-0.55) | 4.87 (-0.06) | 4.25 (-0.69) | 4.02 (-0.92) |
| | 8.61 | -CHO | 4.34 | 4.43 (0.10) | 4.20 (-0.14) | 3.94 (-0.40) | 4.42 (0.09) | 3.81 (-0.53) | 3.60 (-0.74) |
| | 8.60 | -CN | 4.45 | 4.54 (0.09) | 4.28 (-0.16) | 4.04 (-0.40) | 4.47 (0.02) | 3.93 (-0.52) | 3.75 (-0.70) |
| | 8.57 | $-NO_2$ | 4.09 | 4.20 (0.11) | 3.99 (-0.09) | 3.72 (-0.36) | 4.28 (0.19) | 3.50 (-0.58) | 3.25 (-0.83) |
| 4 | 11.11 | $-NH_2$ | 4.50 | 4.60 (0.10) | 4.20 (-0.30) | 3.88 (-0.62) | 4.35 (-0.15) | 3.72 (-0.79) | 3.49 (-1.02) |
| | 11.03 | -OH | 4.61 | 4.71 (0.10) | 4.28 (-0.32) | 3.96 (-0.65) | 4.43 (-0.18) | 3.80 (-0.81) | 3.57 (-1.04) |
| | 10.22 | -Cl | 4.44 | 4.53 (0.09) | 4.17 (-0.27) | 3.85 (-0.59) | 4.32 (-0.12) | 3.68 (-0.76) | 3.45 (-0.99) |
| | 11.06 | -CHO | 3.96 | 4.06 (0.09) | 3.79 (-0.17) | 3.49 (-0.48) | 3.97 (0.00) | 3.32 (-0.64) | 3.09 (-0.87) |
| | 11.05 | -CN | 4.03 | 4.12 (0.08) | 3.85 (-0.19) | 3.56 (-0.47) | 4.00 (-0.03) | 3.41 (-0.62) | 3.21 (-0.82) |
| | 11.03 | $-NO_2$ | 3.76 | 3.86 (0.10) | 3.64 (-0.13) | 3.34 (-0.43) | 3.85 (0.09) | 3.10 (-0.66) | 2.84 (-0.92) |
| 5 | 13.57 | $-NH_2$ | | | 3.83 | 3.47 | | | |
| | 13.50 | -OH | | | 3.9 | 3.53 | | | |
| | 12.69 | -Cl | | | 3.81 | 3.44 | | | |
| | 13.52 | -CHO | | | 3.5 | 3.15 | | | |
| | 13.51 | -CN | | | 3.54 | 3.2 | | | |
| | 13.48 | $-NO_2$ | | | 3.37 | 3.03 | | | |
| 6 | 16.03 | $-NH_2$ | | | 3.56 | 3.17 | | | |
| | 15.96 | -OH | | | 3.61 | 3.22 | | | |
| | 15.15 | -Cl | | | 3.53 | 3.12 | | | |
| | 16.07 | -CHO | | | 3.28 | 2.88 | | | |
| | 15.97 | -CN | | | 3.31 | 2.91 | | | |
| | 15.94 | $-NO_2$ | | | 3.17 | 2.78 | | | |
| 7 | 18.39 | $-NH_2$ | | | 3.34 | 2.9 | | | |
| | 18.42 | -OH | | | 3.39 | 2.97 | | | |
| | 17.61 | -Cl | | | 3.33 | 2.88 | | | |
| | 18.43 | -CHO | | | 3.12 | 2.67 | | | |
| | 18.42 | -CN | | | 3.13 | 2.7 | | | |
| | 18.39 | $-NO_2$ | | | 3.02 | 2.58 | | | |
| 8 | 20.96 | $-NH_2$ | | | 3.18 | 2.7 | | | |
| | 20.88 | -OH | | | 3.21 | 2.74 | | | |
| | 20.08 | -Cl | | | 3.16 | 2.68 | | | |
| | 20.89 | -CHO | | | 2.98 | 2.5 | | | |
| | 20.88 | -CN | | | 2.99 | 2.52 | | | |
| | 20.85 | $-NO_2$ | | | 2.89 | 2.42 | | | |

**Table S2.** Intermolecular CT excitation energy from electron-donating orbital corresponding to $NH_3$ to electron-withdrawing orbitals corresponding to various electron acceptors (eV). Values in parentheses are errors of CT excitation energies from EOM-CCSD.

| *n* | Distance R(Å) | HX | EOM-CCSD (cc-pVTZ) | EOM-CCSD (cc-pVDZ) | LC-BLYP ($\mu$=0.47) | OT-LC-BLYP | HF | B3LYP | BLYP |
|---|---|---|---|---|---|---|---|---|---|
| 2 | 6.19 | $NH_3$ | 11.13 | 11.93 (0.80) | 11.06 (-0.07) | 11.11 (-0.01) | 12.42 (1.29) | 7.09 (-4.04) | 5.47 (-5.66) |
| | 6.12 | $H_2O$ | 11.03 | 11.66 (0.64) | 10.77 (-0.26) | 10.83 (-0.20) | 12.29 (1.26) | 6.77 (-4.26) | 5.09 (-5.93) |
| | 5.29 | HCl | 10.34 | 10.51 (0.17) | 10.18 (-0.16) | 10.24 (-0.10) | 11.65 (1.31) | 5.79 (-4.55) | 4.39 (-5.94) |
| | 6.16 | $CH_2O$ | 9.72 | 9.90 (0.18) | 9.34 (-0.38) | 9.26 (-0.46) | 11.63 (1.91) | 4.41 (-5.31) | 2.55 (-7.17) |
| | 6.14 | HCN | 11.43 | 11.78 (0.34) | 10.97 (-0.46) | 11.05 (-0.39) | 13.09 (1.65) | 6.15 (-5.29) | 4.34 (-7.09) |
| | 6.12 | $HNO_2$ | 8.79 | 8.99 (0.20) | 8.03 (-0.76) | 8.03 (-0.76) | 10.32 (1.53) | 3.24 (-5.55) | 1.56 (-7.23) |
| 3 | 8.65 | $NH_3$ | 11.71 | 12.57 (0.86) | 11.65 (-0.07) | 11.71 (0.00) | 13.05 (1.34) | 7.22 (-4.49) | 5.57 (-6.15) |
| | 8.57 | $H_2O$ | 11.63 | 12.34 (0.70) | 11.37 (-0.26) | 11.50 (-0.14) | 12.95 (1.31) | 6.84 (-4.80) | 5.11 (-6.52) |
| | 7.76 | HCl | 10.91 | 11.18 (0.27) | 10.76 (-0.15) | 10.82 (-0.09) | 12.32 (1.41) | 6.12 (-4.79) | 4.40 (-6.51) |
| | 8.61 | $CH_2O$ | 10.35 | 10.54 (0.18) | 9.93 (-0.42) | 9.93 (-0.42) | 12.23 (1.88) | 4.52 (-5.83) | 2.53 (-7.82) |
| | 8.60 | HCN | 12.02 | 12.36 (0.34) | 11.53 (-0.49) | 11.53 (-0.49) | 13.65 (1.63) | 6.20 (-5.81) | 4.28 (-7.74) |
| | 8.57 | $HNO_2$ | 9.4 | 9.59 (0.19) | 8.65 (-0.75) | 8.65 (-0.75) | 10.96 (1.55) | 3.36 (-6.04) | 1.55 (-7.85) |
| 4 | 11.11 | $NH_3$ | 12.08 | 12.93 (0.85) | 12.02 (-0.06) | 12.08 (0.00) | 13.41 (1.33) | 7.27 (-4.81) | 5.54 (-6.54) |
| | 11.03 | $H_2O$ | 12.02 | 12.71 (0.69) | 11.76 (-0.26) | 11.88 (-0.13) | 13.33 (1.31) | 6.88 (-5.13) | 5.08 (-6.93) |
| | 10.22 | HCl | 11.33 | 11.59 (0.26) | 11.18 (-0.15) | 11.25 (-0.08) | 12.75 (1.42) | 6.19 (-5.14) | 4.37 (-6.96) |
| | 11.06 | $CH_2O$ | 10.71 | 10.90 (0.19) | 10.28 (-0.43) | 10.28 (-0.43) | 12.58 (1.87) | 4.59 (-6.13) | 2.53 (-8.18) |
| | 11.05 | HCN | 12.35 | 12.69 (0.34) | 11.85 (-0.50) | 11.99 (-0.36) | 13.98 (1.63) | 6.25 (-6.10) | 4.26 (-8.10) |
| | 11.03 | $HNO_2$ | 9.75 | 9.94 (0.19) | 8.99 (-0.76) | 8.99 (-0.76) | 11.30 (1.55) | 3.42 (-6.33) | 1.54 (-8.21) |
| 5 | 13.57 | $NH_3$ | 12.3 | 13.15 (0.85) | 12.25 (-0.05) | 12.31 (0.01) | 13.63 (1.33) | 7.30 (-5.00) | 5.52 (-6.78) |
| | 13.50 | $H_2O$ | 12.25 | 12.94 (0.69) | 11.99 (-0.25) | 12.12 (-0.13) | 13.56 (1.31) | 6.91 (-5.34) | 5.06 (-7.19) |
| | 12.69 | HCl | 11.58 | 11.84 (0.25) | 11.44 (-0.14) | 11.51 (-0.08) | 13.01 (1.43) | 6.23 (-5.36) | 4.36 (-7.22) |
| | 13.52 | $CH_2O$ | 10.94 | 11.13 (0.19) | 10.50 (-0.44) | 10.50 (-0.44) | 12.81 (1.87) | 4.63 (-6.31) | 2.53 (-8.41) |
| | 13.51 | HCN | 12.57 | 12.91 (0.34) | 12.07 (-0.51) | 12.21 (-0.36) | 14.20 (1.62) | 6.29 (-6.29) | 4.25 (-8.33) |
| | 13.48 | $HNO_2$ | 9.98 | 10.17 (0.19) | 9.21 (-0.77) | 9.21 (-0.77) | 11.52 (1.55) | 3.47 (-6.51) | 1.54 (-8.44) |
| 6 | 16.03 | $NH_3$ | 12.45 | 13.30 (0.85) | 12.40 (-0.05) | 12.46 (0.01) | 13.78 (1.33) | 7.32 (-5.13) | 5.51 (-6.94) |
| | 15.96 | $H_2O$ | 12.4 | 13.09 (0.69) | 12.15 (-0.25) | 12.28 (-0.12) | 13.72 (1.31) | 6.93 (-5.47) | 5.05 (-7.36) |
| | 15.15 | HCl | 11.76 | 12.01 (0.25) | 11.62 (-0.14) | 11.68 (-0.07) | 13.19 (1.43) | 6.25 (-5.50) | 4.35 (-7.41) |
| | 16.07 | $CH_2O$ | 11.11 | 11.30 (0.19) | 10.67 (-0.44) | 10.67 (-0.44) | 12.98 (1.87) | 4.66 (-6.45) | 2.53 (-8.58) |
| | 15.97 | HCN | 12.73 | 13.07 (0.34) | 12.22 (-0.51) | 12.37 (-0.37) | 14.36 (1.62) | 6.32 (-6.42) | 4.24 (-8.49) |
| | 15.94 | $HNO_2$ | 10.12 | 10.32 (0.20) | 9.31 (-0.81) | 9.31 (-0.81) | 11.63 (1.51) | 3.46 (-6.66) | 1.52 (-8.60) |
| 7 | 18.39 | $NH_3$ | 12.56 | 13.41 (0.85) | 12.51 (-0.05) | 12.57 (0.01) | 13.89 (1.33) | 7.33 (-5.23) | 5.50 (-7.06) |
| | 18.42 | $H_2O$ | 12.52 | 13.21 (0.69) | 12.27 (-0.25) | 12.39 (-0.12) | 13.83 (1.31) | 6.95 (-5.57) | 5.04 (-7.48) |
| | 17.61 | HCl | 11.88 | 12.14 (0.25) | 11.75 (-0.14) | 11.81 (-0.07) | 13.32 (1.43) | 6.28 (-5.61) | 4.35 (-7.54) |
| | 18.43 | $CH_2O$ | 11.23 | 11.41 (0.19) | 10.78 (-0.45) | 10.78 (-0.45) | 13.09 (1.87) | 4.69 (-6.54) | 2.53 (-8.69) |
| | 18.42 | HCN | 12.85 | 13.19 (0.34) | 12.34 (-0.51) | 12.48 (-0.37) | 14.47 (1.62) | 6.34 (-6.51) | 4.24 (-8.61) |
| | 18.39 | $HNO_2$ | 10.24 | 10.44 (0.20) | 9.43 (-0.81) | 9.43 (-0.81) | 11.75 (1.51) | 3.49 (-6.75) | 1.52 (-8.72) |
| 8 | 20.96 | $NH_3$ | 12.64 | 13.49 (0.85) | 12.60 (-0.05) | 12.66 (0.01) | 13.97 (1.33) | 7.34 (-5.30) | 5.49 (-7.16) |
| | 20.88 | $H_2O$ | 12.6 | 13.29 (0.69) | 12.36 (-0.25) | 12.48 (-0.12) | 13.92 (1.31) | 6.96 (-5.65) | 5.03 (-7.57) |
| | 20.08 | HCl | 11.98 | 12.23 (0.25) | 11.84 (-0.14) | 11.91 (-0.07) | 13.41 (1.43) | 6.29 (-5.69) | 4.34 (-7.64) |
| | 20.89 | $CH_2O$ | 11.32 | 11.51 (0.19) | 10.87 (-0.45) | 10.87 (-0.45) | 13.19 (1.87) | 4.71 (-6.61) | 2.54 (-8.78) |
| | 20.88 | HCN | 12.94 | 13.28 (0.34) | 12.43 (-0.51) | 12.57 (-0.37) | 14.56 (1.62) | 6.36 (-6.58) | 4.24 (-8.70) |
| | 20.85 | $HNO_2$ | 10.34 | 10.54 (0.20) | 9.53 (-0.81) | 9.53 (-0.81) | 11.85 (1.51) | 3.51 (-6.83) | 1.53 (-8.81) |

**Table S3.** Intramolecular orbital energy gaps between electron-donating orbital corresponding to $-NH_2$ and electron-withdrawing orbitals corresponding to various electron acceptors (eV).

| *n* | Distance R(Å) | -X | HF | HF (cc-pVDZ) | LC-BLYP ($\mu$=0.47) | OT-LC-BLYP | B3LYP | BLYP |
|---|---|---|---|---|---|---|---|---|
| 2 | 6.19 | $-NH_2$ | 11.25 | 11.27 | 10.06 | 8.90 | 4.83 | 3.34 |
| | 6.12 | -OH | 11.45 | 11.50 | 10.27 | 9.18 | 4.98 | 3.46 |
| | 5.29 | -Cl | 11.16 | 11.30 | 10.12 | 8.96 | 4.88 | 3.39 |
| | 6.16 | -CHO | 10.35 | 10.38 | 9.26 | 8.19 | 4.26 | 2.88 |
| | 6.14 | -CN | 10.50 | 10.55 | 9.46 | 8.38 | 4.42 | 3.03 |
| | 6.12 | $-NO_2$ | 10.14 | 10.17 | 8.99 | 8.04 | 4.00 | 2.65 |
| 3 | 8.65 | $-NH_2$ | 9.86 | 9.92 | 8.93 | 7.61 | 4.01 | 2.69 |
| | 8.57 | -OH | 10.02 | 10.06 | 9.07 | 7.83 | 4.11 | 2.76 |
| | 7.76 | -Cl | 9.85 | 9.90 | 8.93 | 7.62 | 4.02 | 2.70 |
| | 8.61 | -CHO | 9.20 | 9.21 | 8.26 | 7.03 | 3.55 | 2.33 |
| | 8.60 | -CN | 9.29 | 9.32 | 8.39 | 7.16 | 3.66 | 2.42 |
| | 8.57 | $-NO_2$ | 9.01 | 9.02 | 8.04 | 6.85 | 3.36 | 2.17 |
| 4 | 11.11 | $-NH_2$ | 8.99 | 9.01 | 8.16 | 6.73 | 3.46 | 2.25 |
| | 11.03 | -OH | 9.10 | 9.12 | 8.26 | 6.82 | 3.53 | 2.30 |
| | 10.22 | -Cl | 8.96 | 8.98 | 8.14 | 6.72 | 3.45 | 2.25 |
| | 11.06 | -CHO | 8.43 | 8.44 | 7.60 | 6.25 | 3.09 | 1.96 |
| | 11.05 | -CN | 8.50 | 8.51 | 7.69 | 6.35 | 3.16 | 2.03 |
| | 11.03 | $-NO_2$ | 8.26 | 8.26 | 7.40 | 6.11 | 2.93 | 1.84 |
| 5 | 13.57 | $-NH_2$ | | | 7.62 | 6.05 | | |
| | 13.50 | -OH | | | 7.69 | 6.12 | | |
| | 12.69 | -Cl | | | 7.59 | 6.03 | | |
| | 13.52 | -CHO | | | 7.13 | 5.65 | | |
| | 13.51 | -CN | | | 7.19 | 5.72 | | |
| | 13.48 | $-NO_2$ | | | 6.94 | 5.51 | | |
| 6 | 16.03 | $-NH_2$ | | | 7.21 | 5.59 | | |
| | 15.96 | -OH | | | 7.28 | 5.65 | | |
| | 15.15 | -Cl | | | 7.18 | 5.46 | | |
| | 16.07 | -CHO | | | 6.78 | 5.13 | | |
| | 15.97 | -CN | | | 6.82 | 5.19 | | |
| | 15.94 | $-NO_2$ | | | 6.60 | 5.01 | | |
| 7 | 18.39 | $-NH_2$ | | | 6.91 | 5.11 | | |
| | 18.42 | -OH | | | 6.96 | 5.26 | | |
| | 17.61 | -Cl | | | 6.87 | 5.08 | | |
| | 18.43 | -CHO | | | 6.51 | 4.79 | | |
| | 18.42 | -CN | | | 6.55 | 4.84 | | |
| | 18.39 | $-NO_2$ | | | 6.34 | 4.68 | | |
| 8 | 20.96 | $-NH_2$ | | | 6.67 | 4.78 | | |
| | 20.88 | -OH | | | 6.71 | 4.82 | | |
| | 20.08 | -Cl | | | 6.62 | 4.76 | | |
| | 20.89 | -CHO | | | 6.30 | 4.49 | | |
| | 20.88 | -CN | | | 6.33 | 4.53 | | |
| | 20.85 | $-NO_2$ | | | 6.13 | 4.39 | | |

**Table S4.** Intermolecular orbital energy gaps between electron-donating orbital corresponding to $NH_3$ and electron-withdrawing orbitals corresponding to various electron acceptors (eV).

| *n* | Distance R(Å) | HX | HF | HF (cc-pVDZ) | LC-BLYP ($\mu$=0.47) | OT-LC-BLYP | B3LYP | BLYP |
|---|---|---|---|---|---|---|---|---|
| 2 | 6.19 | $NH_3$ | 15.02 | 15.76 | 13.59 | 13.65 | 7.36 | 5.39 |
| | 6.12 | $H_2O$ | 15.00 | 15.56 | 12.93 | 12.99 | 7.64 | 5.07 |
| | 5.29 | HCl | 13.91 | 14.60 | 12.43 | 12.49 | 6.45 | 4.39 |
| | 6.16 | $CH_2O$ | 13.90 | 14.12 | 11.59 | 11.51 | 4.86 | 2.55 |
| | 6.14 | HCN | 15.35 | 15.73 | 13.22 | 13.30 | 6.59 | 4.34 |
| | 6.12 | $HNO_2$ | 12.57 | 12.54 | 10.27 | 10.27 | 3.69 | 1.56 |
| 3 | 8.65 | $NH_3$ | 14.76 | 15.90 | 13.35 | 13.42 | 7.56 | 5.56 |
| | 8.57 | $H_2O$ | 14.71 | 15.64 | 13.14 | 13.26 | 7.18 | 5.11 |
| | 7.76 | HCl | 14.19 | 14.63 | 12.62 | 12.68 | 6.49 | 4.40 |
| | 8.61 | $CH_2O$ | 13.87 | 14.09 | 11.56 | 11.56 | 4.84 | 2.53 |
| | 8.60 | HCN | 15.28 | 15.66 | 13.15 | 13.15 | 6.53 | 4.28 |
| | 8.57 | $HNO_2$ | 12.59 | 12.56 | 10.28 | 10.28 | 3.69 | 1.55 |
| 4 | 11.11 | $NH_3$ | 14.72 | 15.87 | 13.33 | 13.39 | 7.53 | 5.54 |
| | 11.03 | $H_2O$ | 14.68 | 15.60 | 13.11 | 13.23 | 7.15 | 5.08 |
| | 10.22 | HCl | 14.17 | 14.60 | 12.59 | 12.66 | 6.46 | 4.37 |
| | 11.06 | $CH_2O$ | 13.86 | 14.09 | 11.55 | 11.55 | 4.84 | 2.53 |
| | 11.05 | HCN | 15.26 | 15.64 | 13.12 | 13.27 | 6.50 | 4.26 |
| | 11.03 | $HNO_2$ | 12.57 | 12.54 | 10.26 | 10.26 | 3.68 | 1.54 |
| 5 | 13.57 | $NH_3$ | 14.70 | 15.84 | 13.32 | 13.38 | 7.51 | 5.52 |
| | 13.50 | $H_2O$ | 14.65 | 15.57 | 13.09 | 13.21 | 7.13 | 5.06 |
| | 12.69 | HCl | 14.15 | 14.58 | 12.58 | 12.64 | 6.45 | 4.36 |
| | 13.52 | $CH_2O$ | 13.86 | 14.08 | 11.55 | 11.55 | 4.84 | 2.53 |
| | 13.51 | HCN | 15.25 | 15.63 | 13.11 | 13.25 | 6.50 | 4.25 |
| | 13.48 | $HNO_2$ | 12.57 | 12.54 | 10.26 | 10.26 | 3.68 | 1.54 |
| 6 | 16.03 | $NH_3$ | 14.68 | 15.82 | 13.30 | 13.36 | 7.50 | 5.51 |
| | 15.96 | $H_2O$ | 14.63 | 15.55 | 13.07 | 13.20 | 7.11 | 5.05 |
| | 15.15 | HCl | 14.14 | 14.57 | 12.57 | 12.63 | 6.44 | 4.35 |
| | 16.07 | $CH_2O$ | 13.86 | 14.09 | 11.55 | 11.55 | 4.84 | 2.53 |
| | 15.97 | HCN | 15.25 | 15.63 | 13.11 | 13.25 | 6.49 | 4.24 |
| | 15.94 | $HNO_2$ | 12.52 | 12.49 | 10.20 | 10.20 | 3.64 | 1.52 |
| 7 | 18.39 | $NH_3$ | 14.67 | 15.81 | 13.29 | 13.35 | 7.49 | 5.5 |
| | 18.42 | $H_2O$ | 14.62 | 15.54 | 13.06 | 13.19 | 7.1 | 5.04 |
| | 17.61 | HCl | 14.14 | 14.57 | 12.56 | 12.63 | 6.44 | 4.35 |
| | 18.43 | $CH_2O$ | 13.87 | 14.09 | 11.55 | 11.55 | 4.84 | 2.53 |
| | 18.42 | HCN | 15.25 | 15.63 | 13.11 | 13.25 | 6.49 | 4.24 |
| | 18.39 | $HNO_2$ | 12.52 | 12.49 | 10.2 | 10.2 | 3.64 | 1.52 |
| 8 | 20.96 | $NH_3$ | 14.66 | 15.8 | 13.29 | 13.35 | 7.48 | 5.49 |
| | 20.88 | $H_2O$ | 14.61 | 15.53 | 13.05 | 13.18 | 7.1 | 5.03 |
| | 20.08 | HCl | 14.13 | 14.56 | 12.56 | 12.62 | 6.43 | 4.34 |
| | 20.89 | $CH_2O$ | 13.87 | 14.09 | 11.55 | 11.55 | 4.84 | 2.54 |
| | 20.88 | HCN | 15.25 | 15.63 | 13.11 | 13.25 | 6.49 | 4.24 |
| | 20.85 | $HNO_2$ | 12.53 | 12.5 | 10.21 | 10.21 | 3.65 | 1.53 |

**Table S5.** Intramolecular exciton binding energies between electron-donating orbital corresponding to $-NH_2$ and electron-withdrawing orbitals corresponding to various electron acceptors (eV).

| *n* | Distance R(Å) | -X | EOM-CCSD (cc-pVTZ) | EOM-CCSD (cc-pVDZ) | LC-BLYP ($\mu$=0.47) | OT-LC-BLYP | HF | B3LYP | BLYP |
|---|---|---|---|---|---|---|---|---|---|
| 2 | 6.19 | $-NH_2$ | -5.44 | -5.49 | -4.64 | -3.87 | -5.56 | 0.27 | 1.52 |
| | 6.12 | -OH | -5.49 | -5.51 | -4.68 | -3.76 | -5.59 | 0.27 | 1.56 |
| | 5.29 | -Cl | -5.50 | -5.49 | -4.68 | -3.78 | -5.46 | 0.12 | 1.46 |
| | 6.16 | -CHO | -5.47 | -5.40 | -4.46 | -3.59 | -5.26 | 0.24 | 1.42 |
| | 6.14 | -CN | -5.42 | -5.37 | -4.52 | -3.63 | -5.34 | 0.25 | 1.47 |
| | 6.12 | $-NO_2$ | -5.60 | -5.50 | -4.50 | -3.78 | -5.25 | 0.05 | 1.15 |
| 3 | 8.65 | $-NH_2$ | -4.86 | -4.80 | -4.22 | -3.19 | -4.95 | 0.24 | 1.42 |
| | 8.57 | -OH | -4.87 | -4.80 | -4.25 | -3.28 | -5.00 | 0.27 | 1.43 |
| | 7.76 | -Cl | -4.91 | -4.85 | -4.25 | -3.22 | -4.98 | 0.23 | 1.32 |
| | 8.61 | -CHO | -4.86 | -4.78 | -4.07 | -3.09 | -4.77 | 0.25 | 1.27 |
| | 8.60 | -CN | -4.85 | -4.78 | -4.11 | -3.12 | -4.82 | 0.27 | 1.33 |
| | 8.57 | $-NO_2$ | -4.92 | -4.83 | -4.05 | -3.13 | -4.73 | 0.14 | 1.08 |
| 4 | 11.11 | $-NH_2$ | -4.49 | -4.41 | -3.96 | -2.86 | -4.64 | 0.25 | 1.23 |
| | 11.03 | -OH | -4.49 | -4.41 | -3.98 | -2.86 | -4.67 | 0.27 | 1.27 |
| | 10.22 | -Cl | -4.52 | -4.45 | -3.97 | -2.88 | -4.65 | 0.23 | 1.20 |
| | 11.06 | -CHO | -4.47 | -4.38 | -3.81 | -2.77 | -4.47 | 0.23 | 1.13 |
| | 11.05 | -CN | -4.47 | -4.40 | -3.84 | -2.79 | -4.50 | 0.25 | 1.17 |
| | 11.03 | $-NO_2$ | -4.49 | -4.40 | -3.76 | -2.77 | -4.41 | 0.17 | 1.00 |
| 5 | 13.57 | $-NH_2$ | | | -3.78 | -2.58 | | | |
| | 13.50 | -OH | | | -3.80 | -2.59 | | | |
| | 12.69 | -Cl | | | -3.78 | -2.60 | | | |
| | 13.52 | -CHO | | | -3.63 | -2.50 | | | |
| | 13.51 | -CN | | | -3.66 | -2.52 | | | |
| | 13.48 | $-NO_2$ | | | -3.57 | -2.48 | | | |
| 6 | 16.03 | $-NH_2$ | | | -3.66 | -2.43 | | | |
| | 15.96 | -OH | | | -3.67 | -2.43 | | | |
| | 15.15 | -Cl | | | -3.64 | -2.35 | | | |
| | 16.07 | -CHO | | | -3.50 | -2.26 | | | |
| | 15.97 | -CN | | | -3.52 | -2.28 | | | |
| | 15.94 | $-NO_2$ | | | -3.43 | -2.24 | | | |
| 7 | 18.39 | $-NH_2$ | | | -3.56 | -2.21 | | | |
| | 18.42 | -OH | | | -3.57 | -2.30 | | | |
| | 17.61 | -Cl | | | -3.54 | -2.21 | | | |
| | 18.43 | -CHO | | | -3.40 | -2.12 | | | |
| | 18.42 | -CN | | | -3.41 | -2.14 | | | |
| | 18.39 | $-NO_2$ | | | -3.32 | -2.09 | | | |
| 8 | 20.96 | $-NH_2$ | | | -3.49 | -2.08 | | | |
| | 20.88 | -OH | | | -3.50 | -2.09 | | | |
| | 20.08 | -Cl | | | -3.46 | -2.08 | | | |
| | 20.89 | -CHO | | | -3.32 | -1.99 | | | |
| | 20.88 | -CN | | | -3.33 | -2.01 | | | |
| | 20.85 | $-NO_2$ | | | -3.24 | -1.96 | | | |

**Table S6.** Intermolecular exciton binding energies between electron-donating orbital corresponding to $NH_3$ and electron-withdrawing orbitals corresponding to various electron acceptors. (eV).

| *n* | Distance R(Å) | HX | EOM-CCSD (cc-pVTZ) | EOM-CCSD (cc-pVDZ) | LC-BLYP ($\mu$=0.47) | OT-LC-BLYP | HF | B3LYP | BLYP |
|---|---|---|---|---|---|---|---|---|---|
| 2 | 6.19 | $NH_3$ | -3.89 | -3.84 | -2.53 | -2.54 | -2.60 | -0.27 | 0.08 |
| | 6.12 | $H_2O$ | -3.98 | -3.90 | -2.16 | -2.16 | -2.71 | -0.87 | 0.02 |
| | 5.29 | HCl | -3.57 | -4.09 | -2.25 | -2.26 | -2.26 | -0.66 | 0.01 |
| | 6.16 | $CH_2O$ | -4.18 | -4.21 | -2.25 | -2.25 | -2.27 | -0.45 | 0.00 |
| | 6.14 | HCN | -3.92 | -3.95 | -2.25 | -2.25 | -2.27 | -0.45 | 0.00 |
| | 6.12 | $HNO_2$ | -3.78 | -3.55 | -2.24 | -2.24 | -2.25 | -0.45 | 0.00 |
| 3 | 8.65 | $NH_3$ | -3.04 | -3.33 | -1.71 | -1.71 | -1.71 | -0.33 | 0.00 |
| | 8.57 | $H_2O$ | -3.08 | -3.30 | -1.77 | -1.77 | -1.77 | -0.35 | 0.00 |
| | 7.76 | HCl | -3.28 | -3.46 | -1.86 | -1.86 | -1.87 | -0.37 | 0.00 |
| | 8.61 | $CH_2O$ | -3.52 | -3.55 | -1.63 | -1.63 | -1.64 | -0.32 | 0.00 |
| | 8.60 | HCN | -3.27 | -3.31 | -1.62 | -1.62 | -1.63 | -0.32 | 0.00 |
| | 8.57 | $HNO_2$ | -3.18 | -2.97 | -1.62 | -1.62 | -1.63 | -0.32 | 0.00 |
| 4 | 11.11 | $NH_3$ | -2.65 | -2.94 | -1.31 | -1.31 | -1.31 | -0.26 | 0.00 |
| | 11.03 | $H_2O$ | -2.66 | -2.89 | -1.35 | -1.35 | -1.35 | -0.27 | 0.00 |
| | 10.22 | HCl | -2.84 | -3.01 | -1.41 | -1.41 | -1.42 | -0.28 | 0.00 |
| | 11.06 | $CH_2O$ | -3.15 | -3.19 | -1.27 | -1.27 | -1.28 | -0.25 | 0.00 |
| | 11.05 | HCN | -2.90 | -2.95 | -1.27 | -1.27 | -1.28 | -0.25 | 0.00 |
| | 11.03 | $HNO_2$ | -2.83 | -2.61 | -1.27 | -1.27 | -1.28 | -0.25 | 0.00 |
| 5 | 13.57 | $NH_3$ | -2.40 | -2.69 | -1.07 | -1.07 | -1.07 | -0.21 | 0.00 |
| | 13.50 | $H_2O$ | -2.40 | -2.63 | -1.09 | -1.09 | -1.09 | -0.22 | 0.00 |
| | 12.69 | HCl | -2.57 | -2.75 | -1.13 | -1.13 | -1.14 | -0.22 | 0.00 |
| | 13.52 | $CH_2O$ | -2.92 | -2.95 | -1.05 | -1.05 | -1.05 | -0.21 | 0.00 |
| | 13.51 | HCN | -2.67 | -2.72 | -1.04 | -1.04 | -1.05 | -0.21 | 0.00 |
| | 13.48 | $HNO_2$ | -2.60 | -2.38 | -1.05 | -1.05 | -1.05 | -0.21 | 0.00 |
| 6 | 16.03 | $NH_3$ | -2.23 | -2.52 | -0.90 | -0.90 | -0.90 | -0.18 | 0.00 |
| | 15.96 | $H_2O$ | -2.23 | -2.46 | -0.92 | -0.92 | -0.92 | -0.18 | 0.00 |
| | 15.15 | HCl | -2.38 | -2.56 | -0.95 | -0.95 | -0.95 | -0.19 | 0.00 |
| | 16.07 | $CH_2O$ | -2.75 | -2.79 | -0.88 | -0.88 | -0.89 | -0.18 | 0.00 |
| | 15.97 | HCN | -2.51 | -2.56 | -0.89 | -0.89 | -0.89 | -0.18 | 0.00 |
| | 15.94 | $HNO_2$ | -2.40 | -2.17 | -0.89 | -0.89 | -0.89 | -0.18 | 0.00 |
| 7 | 18.39 | $NH_3$ | -2.11 | -2.40 | -0.78 | -0.78 | -0.78 | -0.16 | 0.00 |
| | 18.42 | $H_2O$ | -2.10 | -2.33 | -0.79 | -0.79 | -0.79 | -0.16 | 0.00 |
| | 17.61 | HCl | -2.25 | -2.43 | -0.82 | -0.82 | -0.82 | -0.16 | 0.00 |
| | 18.43 | $CH_2O$ | -2.64 | -2.68 | -0.77 | -0.77 | -0.77 | -0.15 | 0.00 |
| | 18.42 | HCN | -2.40 | -2.44 | -0.77 | -0.77 | -0.77 | -0.15 | 0.00 |
| | 18.39 | $HNO_2$ | -2.28 | -2.05 | -0.77 | -0.77 | -0.77 | -0.15 | 0.00 |
| 8 | 20.96 | $NH_3$ | -2.02 | -2.31 | -0.69 | -0.69 | -0.69 | -0.14 | 0.00 |
| | 20.88 | $H_2O$ | -2.01 | -2.24 | -0.70 | -0.70 | -0.70 | -0.14 | 0.00 |
| | 20.08 | HCl | -2.15 | -2.34 | -0.72 | -0.72 | -0.72 | -0.14 | 0.00 |
| | 20.89 | $CH_2O$ | -2.55 | -2.58 | -0.68 | -0.68 | -0.68 | -0.14 | 0.00 |
| | 20.88 | HCN | -2.31 | -2.35 | -0.68 | -0.68 | -0.68 | -0.14 | 0.00 |
| | 20.85 | $HNO_2$ | -2.19 | -1.96 | -0.68 | -0.68 | -0.68 | -0.14 | 0.00 |

**Table S7.** Orbital energy gap, CT excitation energy and exciton binding energy (eV) of intra- and intermolecular CT excitations from -$NH_2$ ($NH_3$) to -$NO_2$ ($HNO_2$) with $n$=4 in a change in $\mu$ value.

| $\mu$ | Intermolecular CT | | | Intramolecular CT | | | Local | | |
|---|---|---|---|---|---|---|---|---|---|
| | Orbital energy gap | CT excitation energy | Exciton binding energy | Orbital energy gap | CT excitation energy | Exciton binding energy | Orbital energy gap | CT excitation energy | Exciton binding energy |
| 0.00 | 1.54 | 1.54 | 0.00 | 1.84 | 2.84 | 1.00 | 5.35 | 5.63 | 0.28 |
| 0.01 | 1.84 | 1.54 | -0.30 | 2.15 | 2.84 | 0.69 | 5.68 | 5.65 | -0.03 |
| 0.02 | 2.15 | 1.57 | -0.58 | 2.45 | 2.84 | 0.40 | 5.99 | 5.66 | -0.33 |
| 0.03 | 2.45 | 1.64 | -0.81 | 2.73 | 2.85 | 0.12 | 6.29 | 5.66 | -0.64 |
| 0.04 | 2.75 | 1.77 | -0.98 | 3.01 | 2.87 | -0.13 | 6.58 | 5.65 | -0.94 |
| 0.05 | 3.04 | 1.94 | -1.10 | 3.26 | 2.89 | -0.37 | 6.87 | 5.64 | -1.23 |
| 0.06 | 3.34 | 2.16 | -1.18 | 3.50 | 2.92 | -0.58 | 7.14 | 5.62 | -1.52 |
| 0.07 | 3.63 | 2.41 | -1.22 | 3.72 | 2.95 | -0.77 | 7.41 | 5.61 | -1.80 |
| 0.08 | 3.92 | 2.67 | -1.25 | 3.93 | 2.98 | -0.95 | 7.67 | 5.59 | -2.07 |
| 0.09 | 4.20 | 2.94 | -1.26 | 4.13 | 3.01 | -1.12 | 7.92 | 5.58 | -2.34 |
| 0.10 | 4.48 | 3.21 | -1.27 | 4.31 | 3.04 | -1.28 | 8.16 | 5.56 | -2.60 |
| 0.11 | 4.75 | 3.48 | -1.27 | 4.49 | 3.06 | -1.42 | 8.40 | 5.55 | -2.85 |
| 0.12 | 5.02 | 3.75 | -1.27 | 4.65 | 3.09 | -1.56 | 8.63 | 5.54 | -3.08 |
| 0.13 | 5.27 | 4.00 | -1.27 | 4.81 | 3.11 | -1.69 | 8.85 | 5.54 | -3.31 |
| 0.14 | 5.53 | 4.26 | -1.27 | 4.95 | 3.14 | -1.82 | 9.06 | 5.53 | -3.53 |
| 0.15 | 5.77 | 4.50 | -1.27 | 5.10 | 3.16 | -1.94 | 9.27 | 5.53 | -3.74 |
| 0.16 | 6.01 | 4.74 | -1.27 | 5.23 | 3.18 | -2.05 | 9.47 | 5.54 | -3.93 |
| 0.17 | 6.24 | 4.97 | -1.27 | 5.36 | 3.20 | -2.16 | 9.67 | 5.55 | -4.12 |
| 0.18 | 6.46 | 5.19 | -1.27 | 5.48 | 3.22 | -2.26 | 9.85 | 5.56 | -4.29 |
| 0.19 | 6.68 | 5.41 | -1.27 | 5.60 | 3.24 | -2.36 | 10.03 | 5.57 | -4.46 |
| 0.20 | 6.89 | 5.62 | -1.27 | 5.71 | 3.26 | -2.45 | 10.21 | 5.59 | -4.62 |
| 0.21 | 7.09 | 5.82 | -1.27 | 5.82 | 3.28 | -2.53 | 10.38 | 5.61 | -4.77 |
| 0.22 | 7.28 | 6.01 | -1.27 | 5.92 | 3.30 | -2.62 | 10.54 | 5.64 | -4.91 |
| 0.23 | 7.47 | 6.20 | -1.27 | 6.01 | 3.32 | -2.70 | 10.70 | 5.67 | -5.04 |
| 0.24 | 7.65 | 6.38 | -1.27 | 6.11 | 3.34 | -2.77 | 10.85 | 5.69 | -5.16 |
| 0.25 | 7.82 | 6.55 | -1.27 | 6.20 | 3.35 | -2.84 | 11.00 | 5.73 | -5.27 |
| 0.26 | 7.99 | 6.72 | -1.27 | 6.28 | 3.37 | -2.91 | 11.14 | 5.76 | -5.38 |
| 0.27 | 8.15 | 6.88 | -1.27 | 6.36 | 3.39 | -2.97 | 11.28 | 5.80 | -5.48 |
| 0.28 | 8.30 | 7.03 | -1.27 | 6.44 | 3.40 | -3.03 | 11.41 | 5.83 | -5.58 |
| 0.29 | 8.45 | 7.18 | -1.27 | 6.51 | 3.42 | -3.09 | 11.54 | 5.87 | -5.67 |
| 0.30 | 8.59 | 7.32 | -1.27 | 6.58 | 3.44 | -3.15 | 11.66 | 5.91 | -5.75 |
| 0.31 | 8.72 | 7.45 | -1.27 | 6.65 | 3.45 | -3.20 | 11.78 | 5.95 | -5.83 |
| 0.32 | 8.85 | 7.58 | -1.27 | 6.72 | 3.47 | -3.25 | 11.89 | 5.99 | -5.90 |
| 0.33 | 8.98 | 7.70 | -1.27 | 6.78 | 3.48 | -3.30 | 12.00 | 6.03 | -5.97 |
| 0.34 | 9.09 | 7.82 | -1.27 | 6.84 | 3.49 | -3.34 | 12.11 | 6.07 | -6.03 |
| 0.35 | 9.21 | 7.94 | -1.27 | 6.89 | 3.51 | -3.38 | 12.21 | 6.11 | -6.09 |
| 0.36 | 9.32 | 8.05 | -1.27 | 6.95 | 3.52 | -3.42 | 12.30 | 6.15 | -6.15 |
| 0.37 | 9.42 | 8.15 | -1.27 | 7.00 | 3.53 | -3.46 | 12.40 | 6.20 | -6.20 |
| 0.38 | 9.52 | 8.25 | -1.27 | 7.05 | 3.55 | -3.50 | 12.49 | 6.24 | -6.25 |

| | | | | | | | | | |
|---|---|---|---|---|---|---|---|---|---|
| 0.39 | 9.62 | 8.35 | -1.27 | 7.09 | 3.56 | -3.53 | 12.58 | 6.28 | -6.30 |
| 0.40 | 9.71 | 8.44 | -1.27 | 7.14 | 3.57 | -3.57 | 12.66 | 6.32 | -6.34 |
| 0.41 | 9.80 | 8.53 | -1.27 | 7.18 | 3.58 | -3.60 | 12.74 | 6.35 | -6.39 |
| 0.42 | 9.89 | 8.61 | -1.27 | 7.22 | 3.59 | -3.63 | 12.82 | 6.39 | -6.42 |
| 0.43 | 9.97 | 8.69 | -1.27 | 7.26 | 3.60 | -3.66 | 12.89 | 6.43 | -6.46 |
| 0.44 | 10.05 | 8.77 | -1.27 | 7.30 | 3.61 | -3.69 | 12.96 | 6.47 | -6.50 |
| 0.45 | 10.12 | 8.85 | -1.27 | 7.33 | 3.62 | -3.71 | 13.03 | 6.50 | -6.53 |
| 0.46 | 10.19 | 8.92 | -1.27 | 7.37 | 3.63 | -3.74 | 13.10 | 6.54 | -6.56 |
| 0.47 | 10.26 | 8.99 | -1.27 | 7.40 | 3.64 | -3.76 | 13.16 | 6.57 | -6.59 |
| 0.48 | 10.33 | 9.05 | -1.27 | 7.43 | 3.64 | -3.79 | 13.22 | 6.60 | -6.62 |
| 0.49 | 10.39 | 9.12 | -1.27 | 7.46 | 3.65 | -3.81 | 13.28 | 6.64 | -6.64 |
| 0.50 | 10.45 | 9.18 | -1.27 | 7.49 | 3.66 | -3.83 | 13.34 | 6.67 | -6.67 |

**Table S8.** Molecular orbitals corresponding to intramolecular CT excitations from -$NH_2$ and various electron acceptor. (H is HOMO and L is LUMO)

| *n* | -X | EOM-CCSD (cc-pVTZ) | EOM-CCSD (cc-pVDZ) | LC-BLYP ($\mu$=0.47) | OT-LC-BLYP | HF | B3LYP | BLYP |
|---|---|---|---|---|---|---|---|---|
| 2 | -$NH_2$ | H → L +3 | H → L | H → L | H → L | H → L +3 | H → L | H → L |
| | -OH | H → L +2 | H → L | H → L | H → L | H → L +2 | H → L | H → L |
| | -Cl | H → L | H → L | H → L | H → L | H → L | H → L | H → L |
| | -CHO | H → L | H → L | H → L | H → L | H → L | H → L | H -1 → L |
| | -CN | H → L | H → L | H → L | H → L | H → L | H → L | H → L |
| | -$NO_2$ | H → L | H → L | H → L | H → L | H → L | H → L | H → L |
| 3 | -$NH_2$ | H → L | H → L | H → L | H → L | H → L | H → L | H → L |
| | -OH | H → L | H → L | H → L | H → L | H → L | H → L | H → L |
| | -Cl | H → L | H → L | H → L | H → L | H → L | H → L | H → L |
| | -CHO | H → L | H → L | H → L | H → L | H → L | H → L | H → L |
| | -CN | H → L | H → L | H → L | H → L | H → L | H → L | H → L |
| | -$NO_2$ | H → L | H → L | H → L | H → L | H → L | H → L | H → L |
| 4 | -$NH_2$ | H → L | H → L | H → L | H → L | H → L | H → L | H → L |
| | -OH | H → L | H → L | H → L | H → L | H → L | H → L | H → L |
| | -Cl | H → L | H → L | H → L | H → L | H → L | H → L | H → L |
| | -CHO | H → L | H → L | H → L | H → L | H → L | H → L | H → L |
| | -CN | H → L | H → L | H → L | H → L | H → L | H → L | H → L |
| | -$NO_2$ | H → L | H → L | H → L | H → L | H → L | H → L | H → L |
| 5 | -$NH_2$ | | | H → L | H → L | | | |
| | -OH | | | H → L | H → L | | | |
| | -Cl | | | H → L | H → L | | | |
| | -CHO | | | H → L | H → L | | | |
| | -CN | | | H → L | H → L | | | |
| | -$NO_2$ | | | H → L | H → L | | | |
| 6 | -$NH_2$ | | | H → L | H → L | | | |
| | -OH | | | H → L | H → L | | | |
| | -Cl | | | H → L | H → L | | | |
| | -CHO | | | H → L | H → L | | | |
| | -CN | | | H → L | H → L | | | |
| | -$NO_2$ | | | H → L | H → L | | | |
| 7 | -$NH_2$ | | | H → L | H → L | | | |
| | -OH | | | H → L | H → L | | | |
| | -Cl | | | H → L | H → L | | | |
| | -CHO | | | H → L | H → L | | | |
| | -CN | | | H → L | H → L | | | |
| | -$NO_2$ | | | H → L | H → L | | | |
| 8 | -$NH_2$ | | | H → L | H → L | | | |
| | -OH | | | H → L | H → L | | | |
| | -Cl | | | H → L | H → L | | | |
| | -CHO | | | H → L | H → L | | | |
| | -CN | | | H → L | H → L | | | |
| | -$NO_2$ | | | H → L | H → L | | | |

**Table S9.** Molecular orbitals corresponding to intramolecular CT excitations from $NH_3$ and various electron acceptor. (H is HOMO and L is LUMO)

| n | HX | EOM-CCSD (cc-pVTZ) | EOM-CCSD (cc-pVDZ) | LC-BLYP ($\mu$=0.47) | OT-LC-BLYP | HF | B3LYP | BLYP |
|---|---|---|---|---|---|---|---|---|
| 2 | $NH_3$ | H → L +1 | H → L +1 | H → L +1 | H → L +1 | H → L +1 | H → L +1 | H → L |
| | $H_2O$ | H → L +1 | H → L | H → L | H → L | H → L +1 | H → L +1 | H → L |
| | HCl | H → L | H → L | H → L | H → L | H → L | H → L | H → L |
| | $CH_2O$ | H → L | H → L | H → L | H → L | H → L | H → L | H → L |
| | HCN | H → L +3 | H → L +2 | H → L +2 | H → L +2 | H → L +3 | H → L +1 | H → L +1 |
| | $HNO_2$ | H → L | H → L | H → L | H → L | H → L | H → L | H → L |
| 3 | $NH_3$ | H → L | H → L | H → L | H → L | H → L | H → L | H → L |
| | $H_2O$ | H → L | H → L | H → L | H → L | H → L | H → L | H → L |
| | HCl | H → L | H → L | H → L | H → L | H → L | H → L | H → L |
| | $CH_2O$ | H → L | H → L | H → L | H → L | H → L | H → L | H → L |
| | HCN | H → L +3 | H → L +2 | H → L +1 | H → L +1 | H → L +3 | H → L +1 | H → L +1 |
| | $HNO_2$ | H → L | H → L | H → L | H → L | H → L | H → L | H → L |
| 4 | $NH_3$ | H → L | H → L | H → L | H → L | H → L | H → L | H → L |
| | $H_2O$ | H → L | H → L | H → L | H → L | H → L | H → L | H → L |
| | HCl | H → L | H → L | H → L | H → L | H → L | H → L | H → L |
| | $CH_2O$ | H → L | H → L | H → L | H → L | H → L | H → L | H → L |
| | HCN | H → L +3 | H → L +1 | H → L +1 | H → L +1 | H → L +3 | H → L +1 | H → L +1 |
| | $HNO_2$ | H → L | H → L | H → L | H → L | H → L | H → L | H → L |
| 5 | $NH_3$ | H → L | H → L | H → L | H → L | H → L | H → L | H → L |
| | $H_2O$ | H → L | H → L | H → L | H → L | H → L | H → L | H → L |
| | HCl | H → L | H → L | H → L | H → L | H → L | H → L | H → L |
| | $CH_2O$ | H → L | H → L | H → L | H → L | H → L | H → L | H → L |
| | HCN | H → L +3 | H → L +1 | H → L +1 | H → L +1 | H → L +3 | H → L +1 | H → L +1 |
| | $HNO_2$ | H → L | H → L | H → L | H → L | H → L | H → L | H → L |
| 6 | $NH_3$ | H → L | H → L | H → L | H → L | H → L | H → L | H → L |
| | $H_2O$ | H → L | H → L | H → L | H → L | H → L | H → L | H → L |
| | HCl | H → L | H → L | H → L | H → L | H → L | H → L | H → L |
| | $CH_2O$ | H → L | H → L | H → L | H → L | H → L | H → L | H → L |
| | HCN | H → L +3 | H → L +1 | H → L +1 | H → L +1 | H → L +3 | H → L +1 | H → L +1 |
| | $HNO_2$ | H → L | H → L | H → L | H → L | H → L | H → L | H → L |
| 7 | $NH_3$ | H → L | H → L | H → L | H → L | H → L | H → L | H → L |
| | $H_2O$ | H → L | H → L | H → L | H → L | H → L | H → L | H → L |
| | HCl | H → L | H → L | H → L | H → L | H → L | H → L | H → L |
| | $CH_2O$ | H → L | H → L | H → L | H → L | H → L | H → L | H → L |
| | HCN | H → L +3 | H → L +1 | H → L +1 | H → L +1 | H → L +3 | H → L +1 | H → L +1 |
| | $HNO_2$ | H → L | H → L | H → L | H → L | H → L | H → L | H → L |
| 8 | $NH_3$ | H → L | H → L | H → L | H → L | H → L | H → L | H → L |
| | $H_2O$ | H → L | H → L | H → L | H → L | H → L | H → L | H → L |
| | HCl | H → L | H → L | H → L | H → L | H → L | H → L | H → L |
| | $CH_2O$ | H → L | H → L | H → L | H → L | H → L | H → L | H → L |
| | HCN | H → L +3 | H → L +1 | H → L +1 | H → L +1 | H → L +3 | H → L +1 | H → L +1 |
| | $HNO_2$ | H → L | H → L | H → L | H → L | H → L | H → L | H → L |

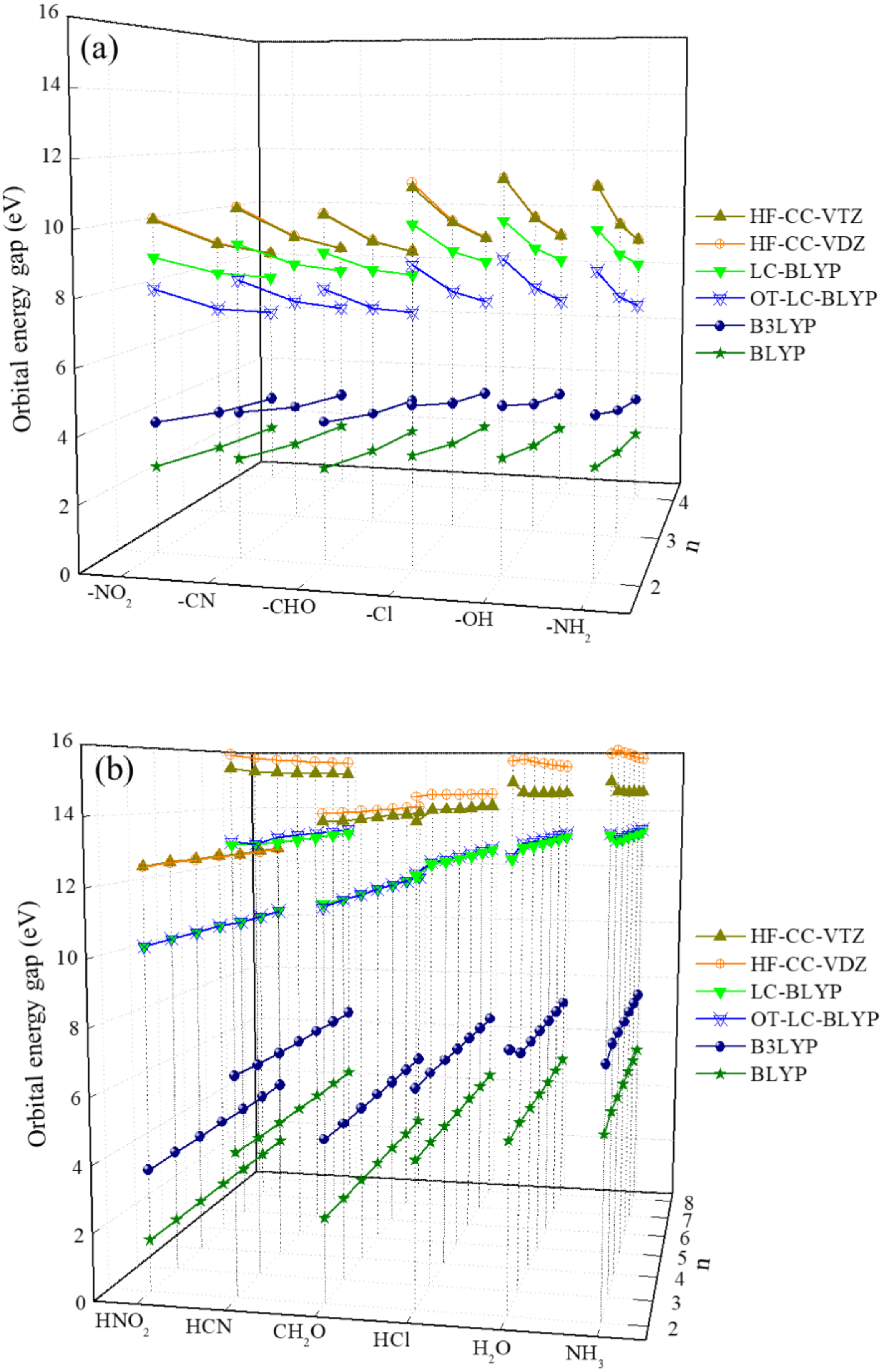


**Figure S1.** Orbital energy gaps corresponding to (a) intramolecular and (b) intermolecular CT excitations between electron-donating and electron-withdrawing orbitals calculated using HF, LC-BLYP, OT-LC-BLYP, B3LYP and BLYP.

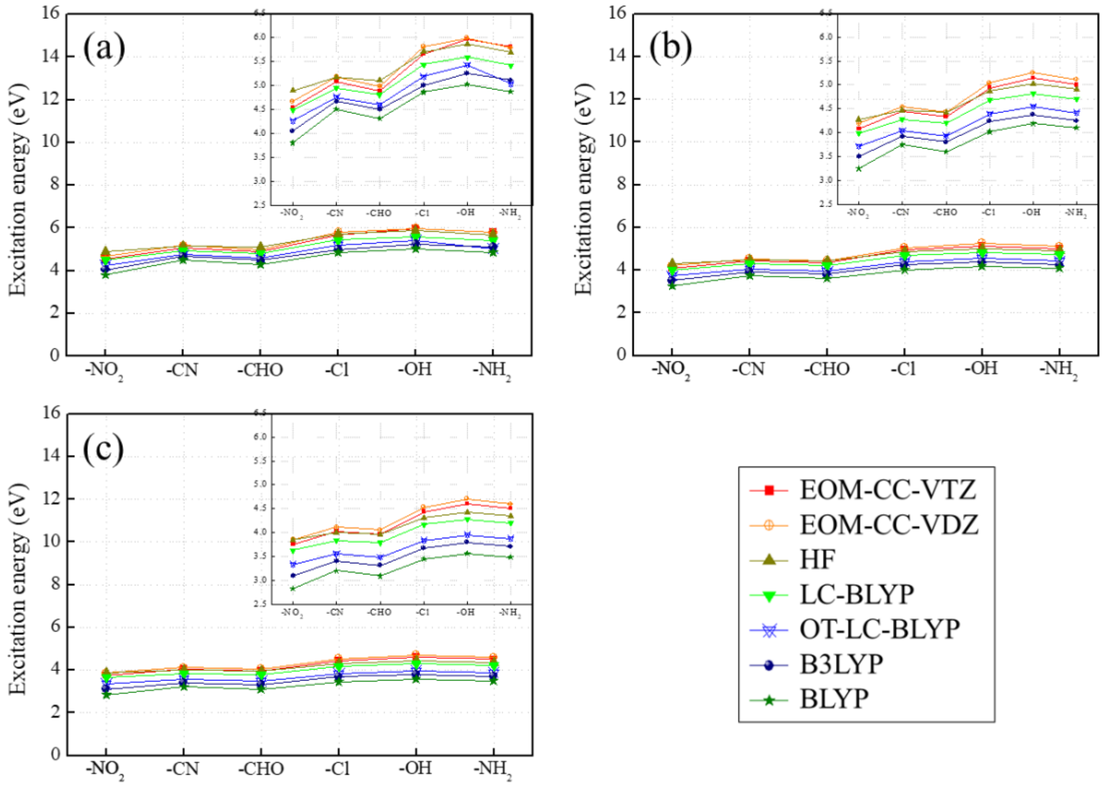


**Figure S2.** Intramolecular CT excitation energies from electron-donating to electron-withdrawing orbitals with (a) *n*=2, (b) *n*=3, and (c) *n*=4 calculated using EOM-CCSD, HF, LC-BLYP, OT-LC-BLYP, B3LYP and BLYP.

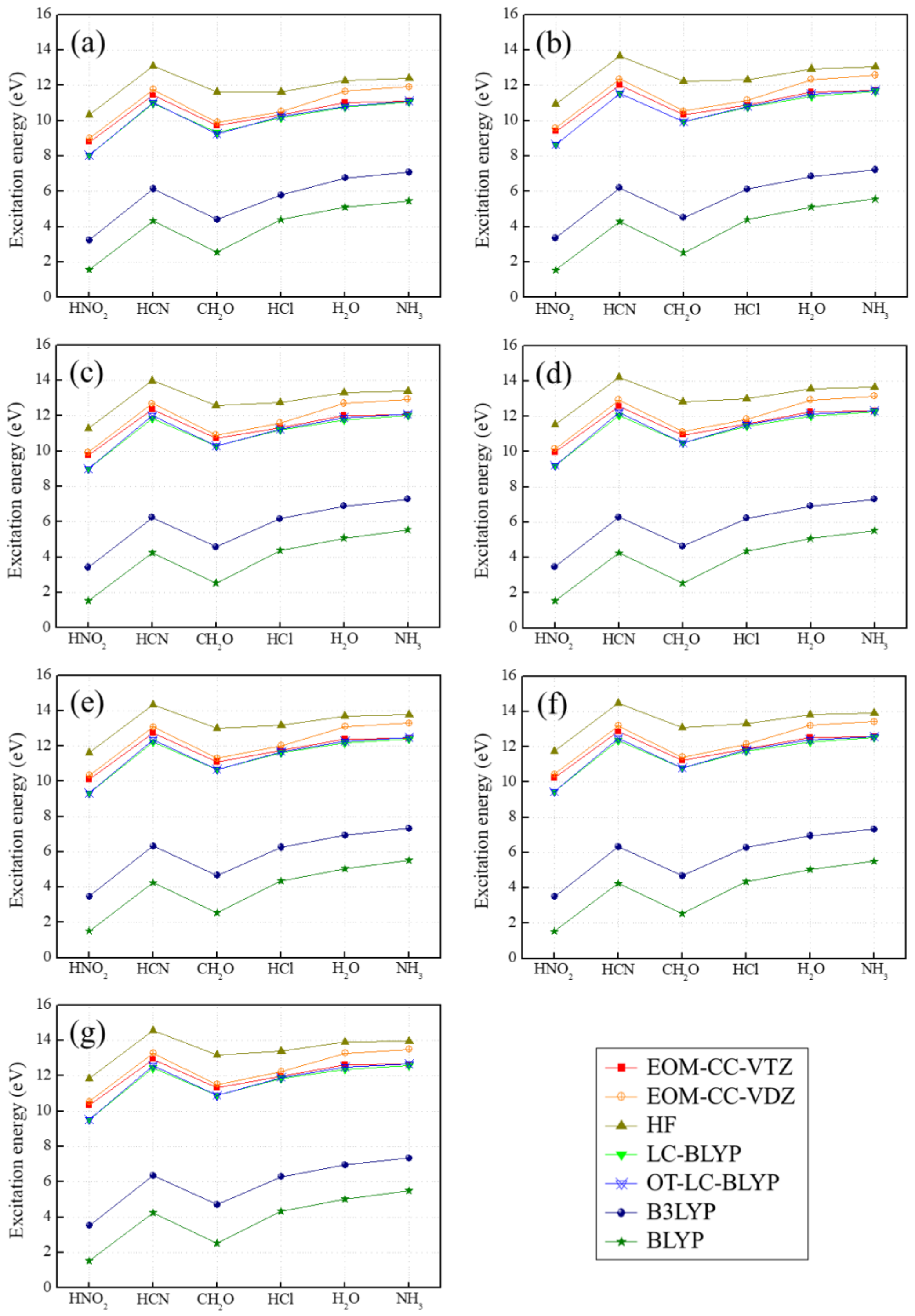


**Figure S3.** Intermolecular CT excitation energies from electron-donating to electron-withdrawing orbitals with (a) $n$=2, (b) $n$=3, (c) $n$=4, (d) $n$=5, (e) $n$=6, (f) $n$=7, and (g) $n$=8 calculated using EOM-CCSD, HF, LC-BLYP, OT-LC-BLYP, B3LYP and BLYP.

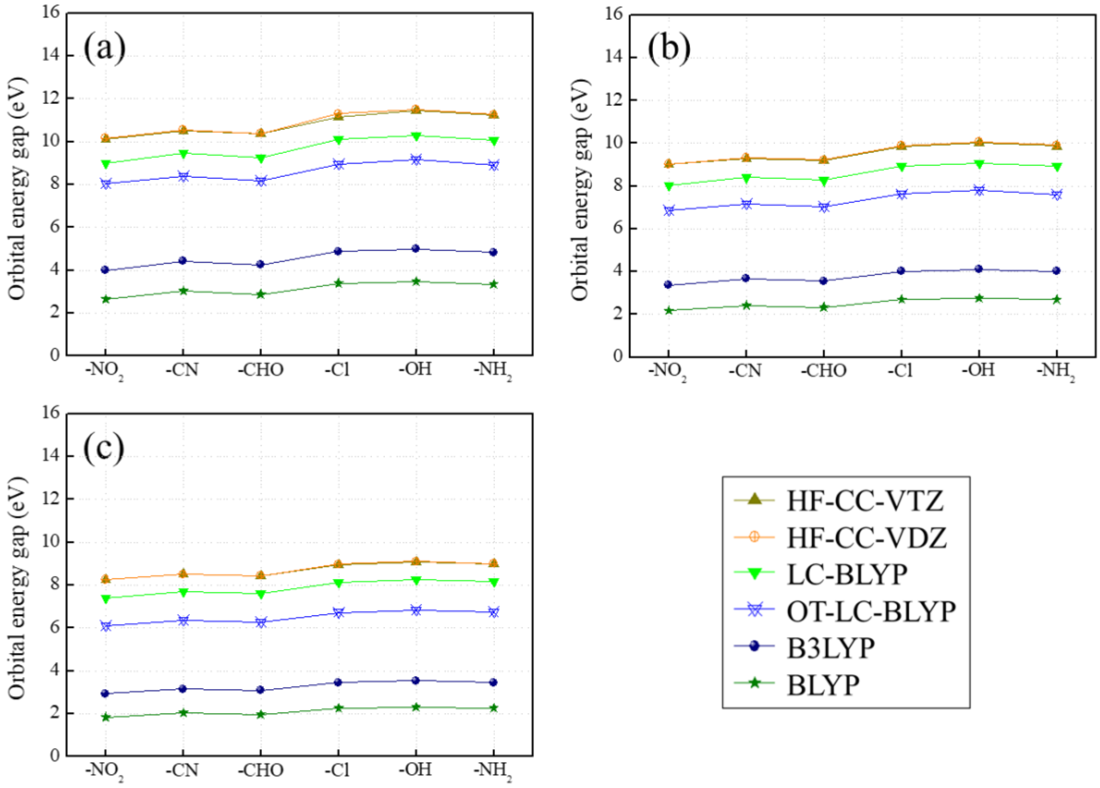


**Figure S4.** Orbital energy gaps between electron-donating and electron-withdrawing orbitals in intramolecular CT excitations with (a) *n*=2, (b) *n*=3, and (c) *n*=4 calculated using HF, LC-BLYP, OT-LC-BLYP, B3LYP and BLYP.

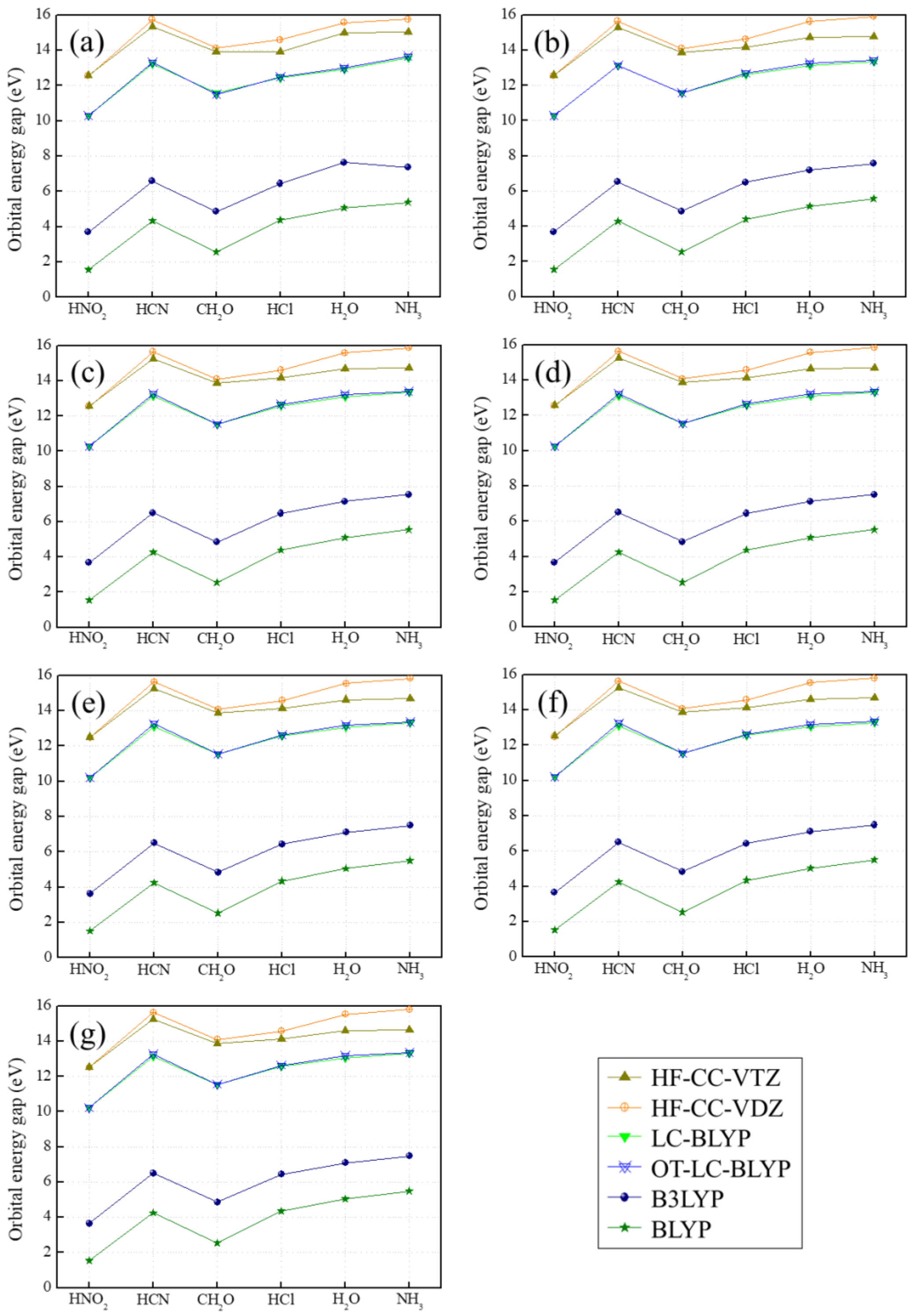


**Figure S5.** Orbital energy gaps between electron-donating and electron-withdrawing orbitals of intermolecular CT excitations with (a) $n$=2, (b) $n$=3, (c) $n$=4, (d) $n$=5, (e) $n$=6, (f) $n$=7, and (g) $n$=8 calculated using HF, LC-BLYP, OT-LC-BLYP, B3LYP and BLYP.

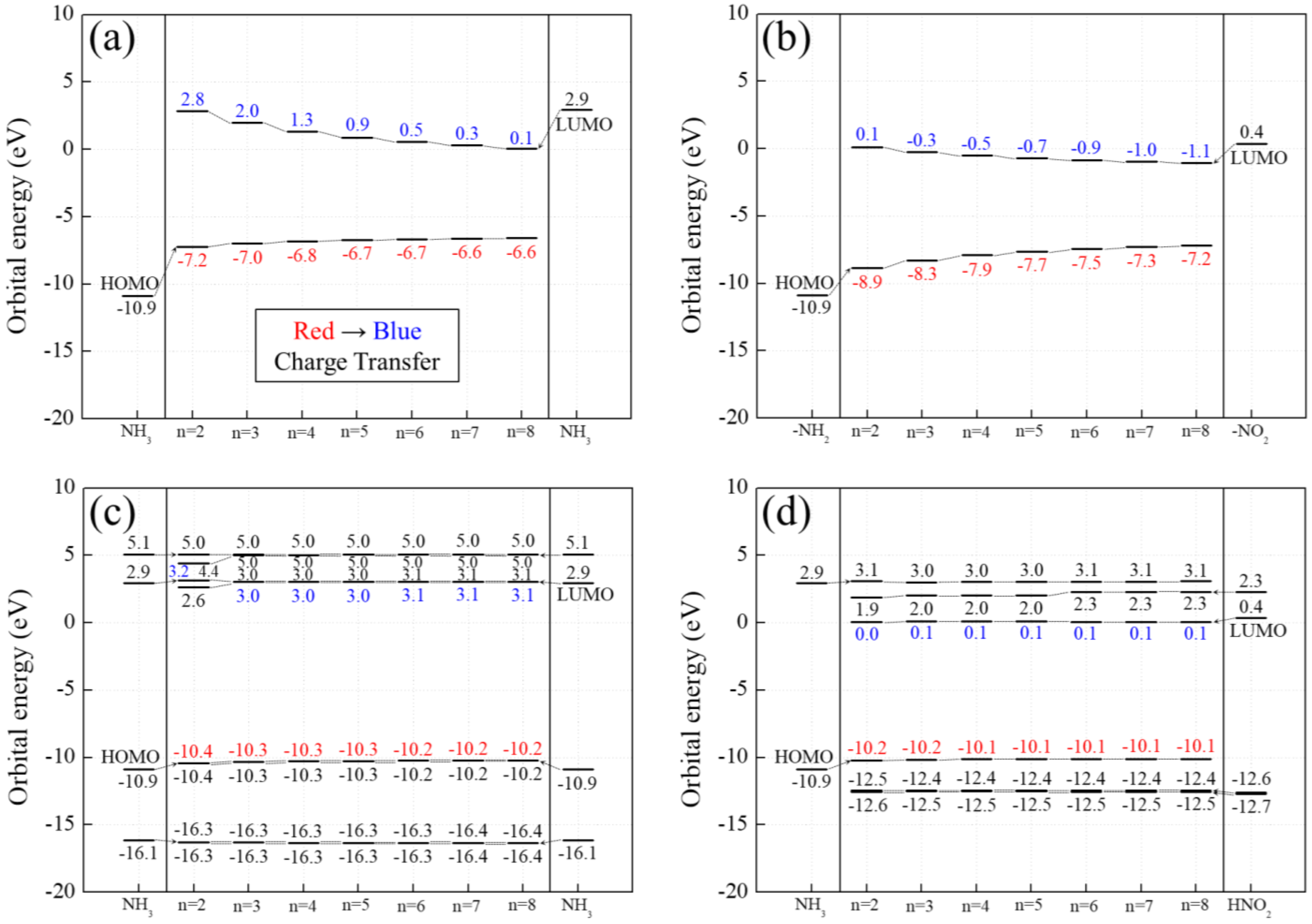


**Figure S6.** Intramolecular HOMO and LUMO energies of (a) H2N–(CH=CH)n–NH2 and (b) H2N–(CH=CH)n–NO2 and intermolecular HOMO and LUMO energies of (c) H2N–H……H–NH2 and (d) H2N–H……H–NO2 with regard to n calculated using LC-BLYP. The HOMO and LUMO energies of NH3 molecule in (a) and (c) and the HOMO energy of NH3 and the LUMO of HNO2 in (b) and (d) are displayed in both sides.

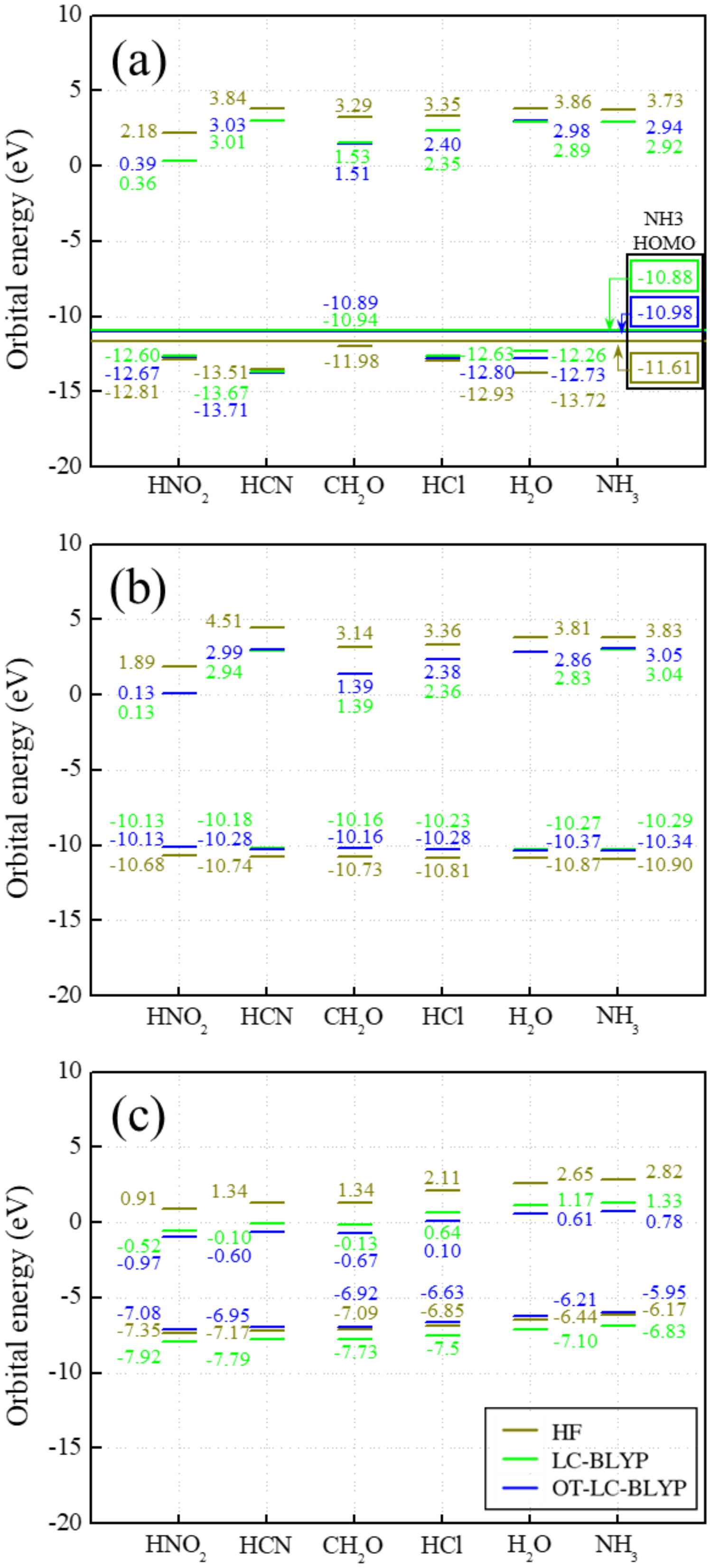


**Figure S7.** (a) HOMO and LUMO energies of electron acceptors as single molecule of (HX) (b) HOMO and LUMO energies of their combination with NH3 molecule for intermolecular CT excitations [H2N–H……H–X] (n=4), (c) HOMO and LUMO energies for intramolecular CT excitations [H2N–(CH=CH)n–NO2] (n=4).

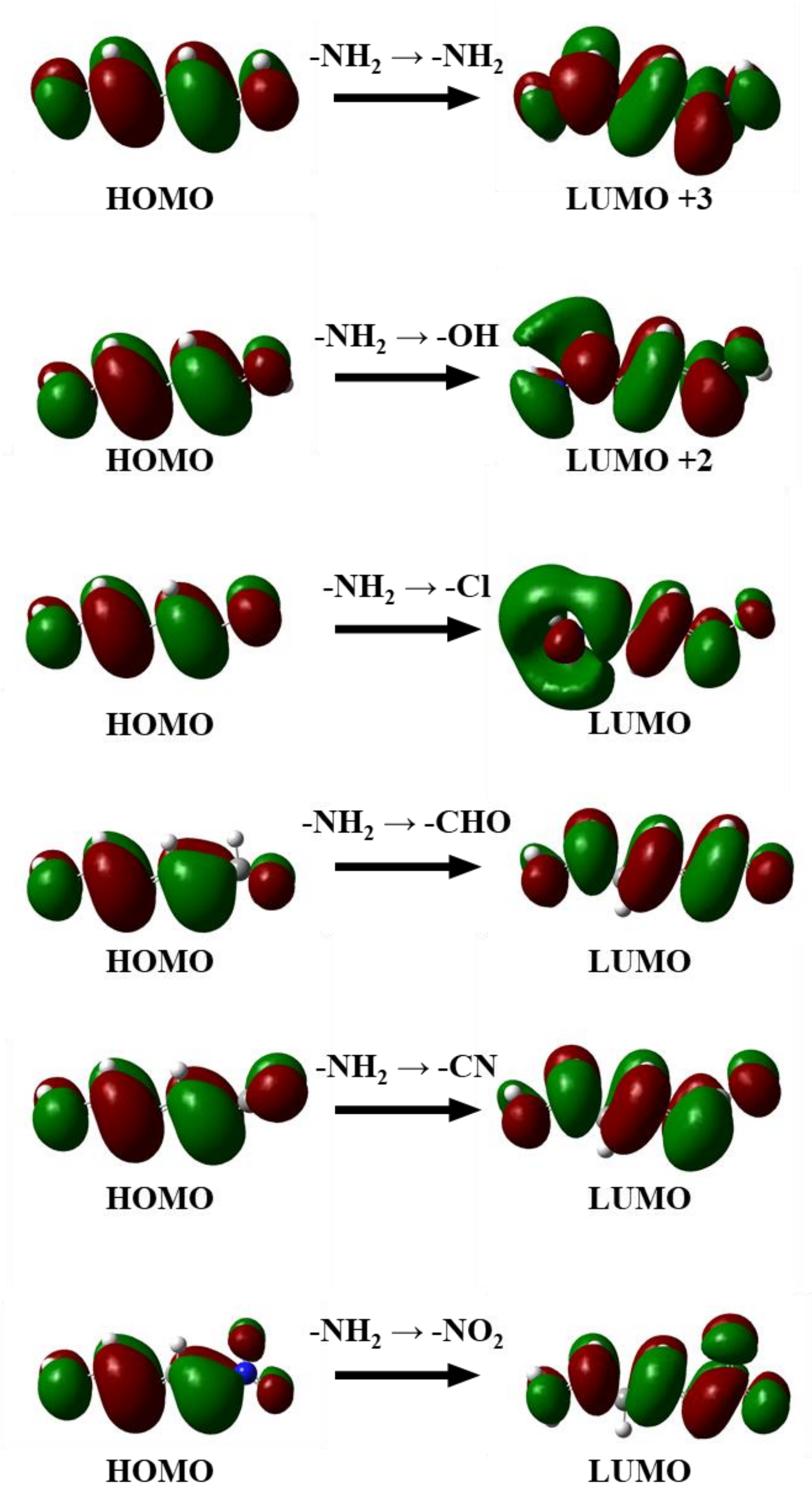


**Figure S8.** MO shapes of intramolecular CT excitations from -$NH_2$ to various electron acceptors with $n$=2 calculated using HF.

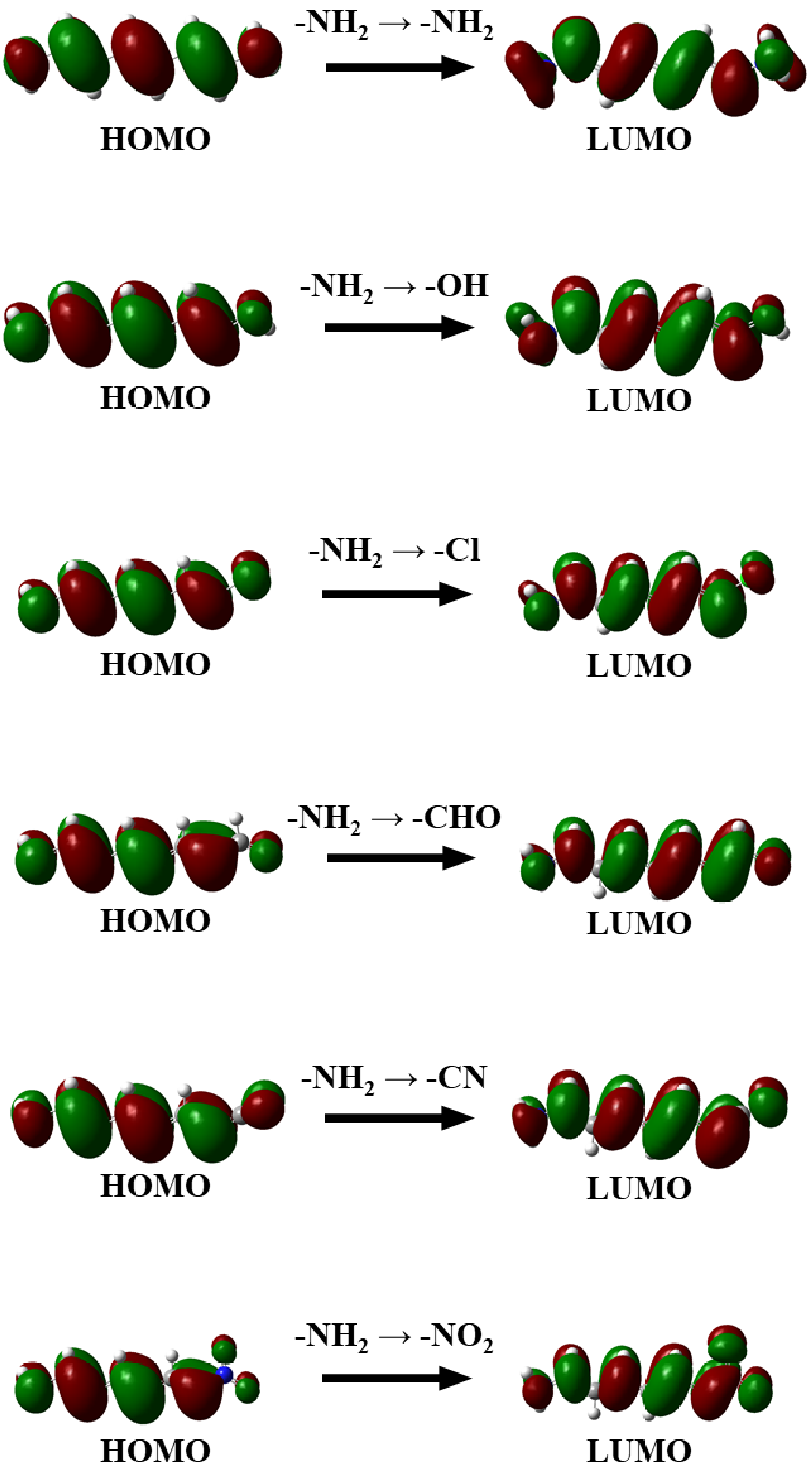


**Figure S9.** MO shapes of intramolecular CT excitations from $-NH_2$ to various electron acceptors with $n$=3 calculated using HF.

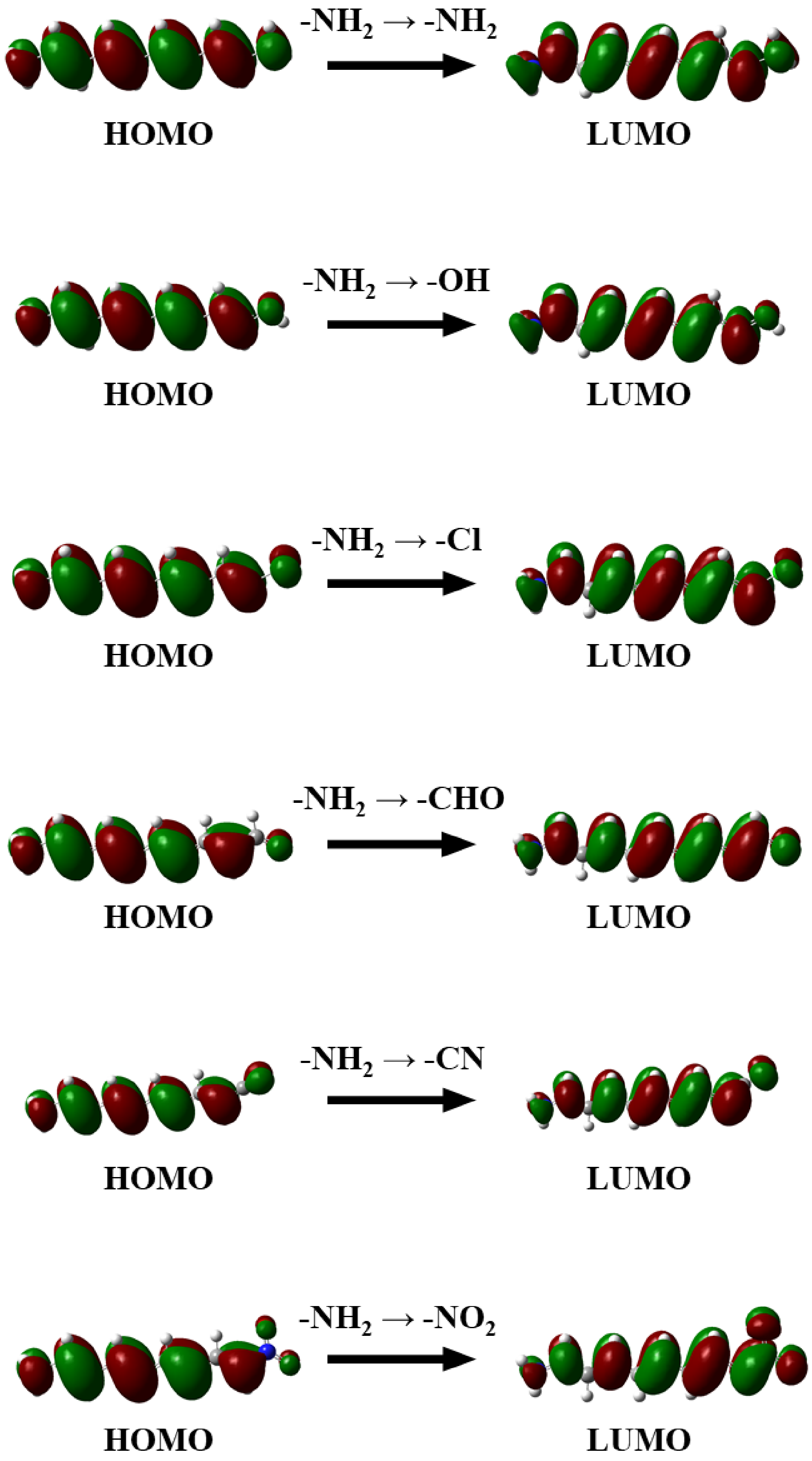


**Figure S10.** MO shapes of intramolecular CT excitations from $-NH_2$ to various electron acceptors with $n$=4 calculated using HF.

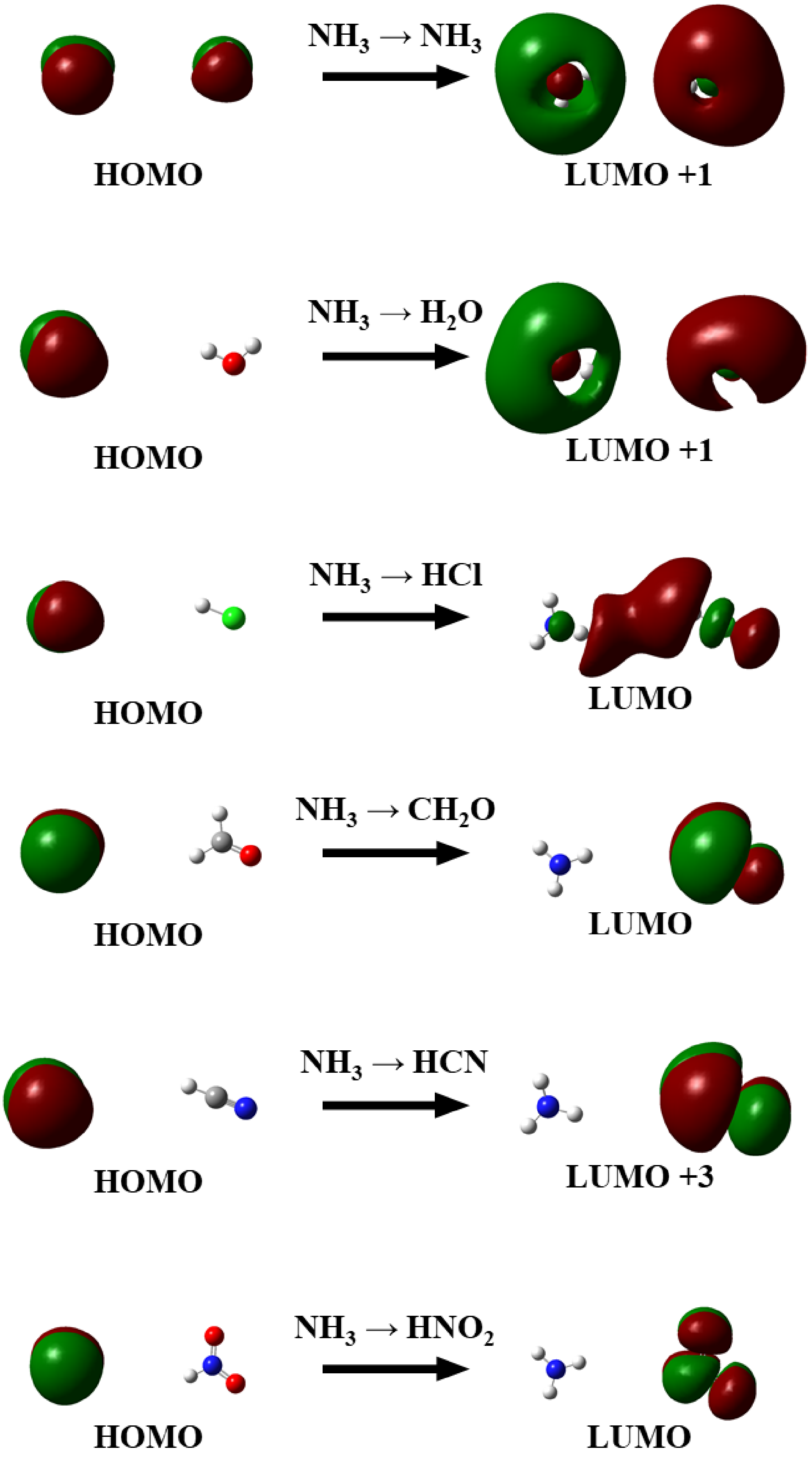


**Figure S11.** MO shapes of intermolecular CT excitations from $NH_3$ to various electron acceptors with $n$=2 calculated using HF.

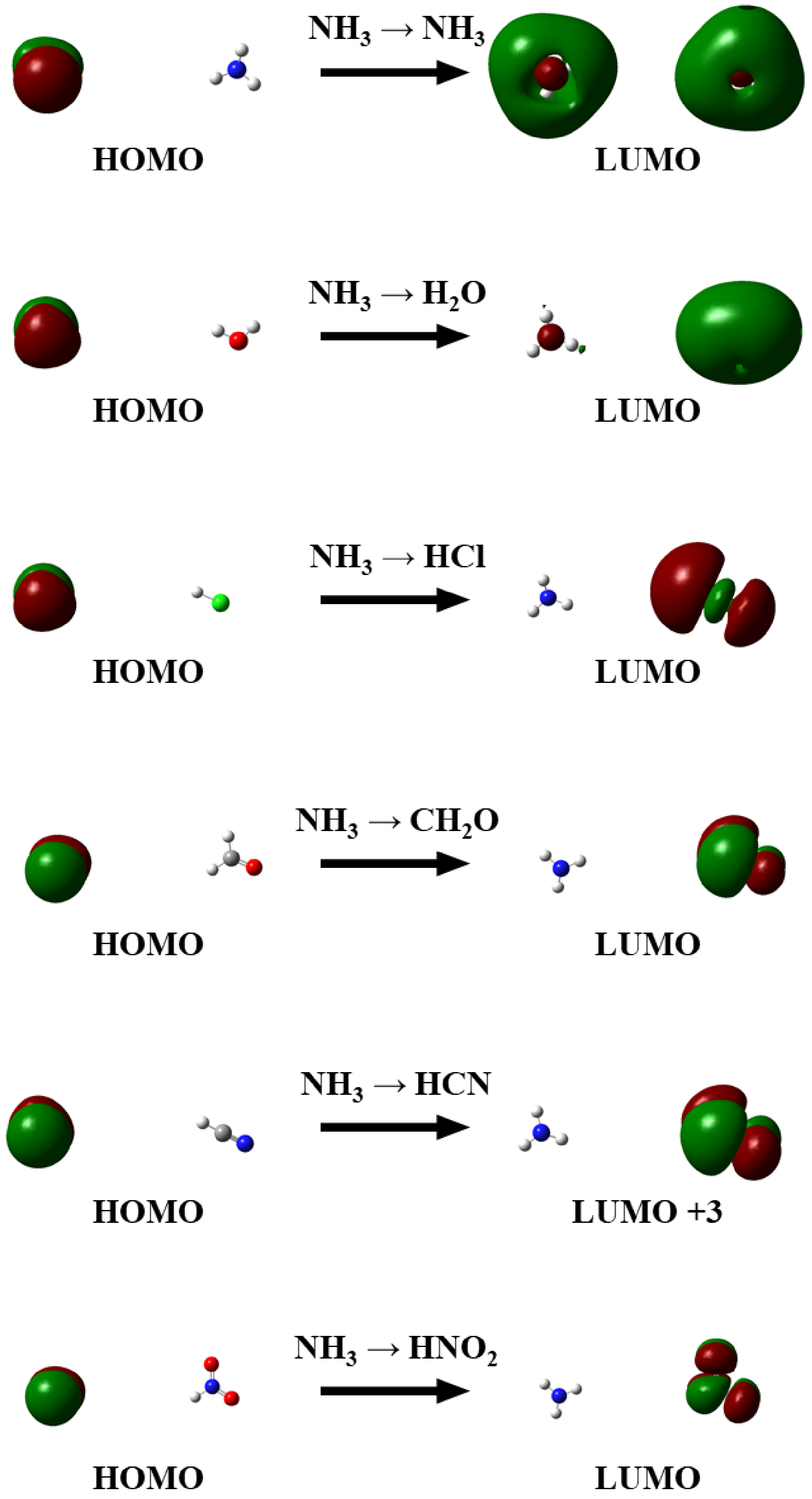


**Figure S12.** MO shapes of intermolecular CT excitations from $NH_3$ to various electron acceptors with *n*=3 calculated using HF.

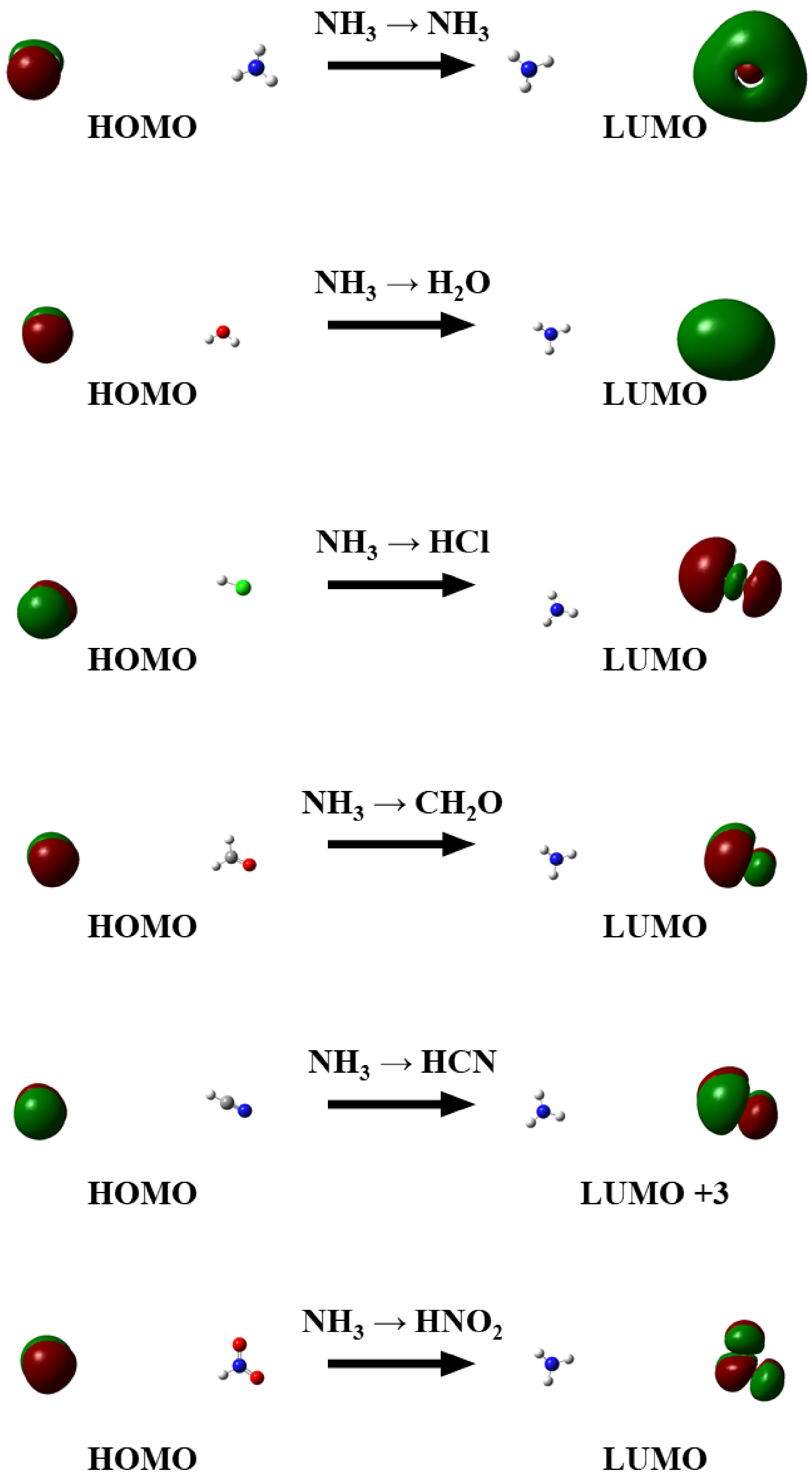


**Figure S13.** MO shapes of intermolecular CT excitations from $NH_3$ to various electron acceptors with $n$=4 calculated using HF.

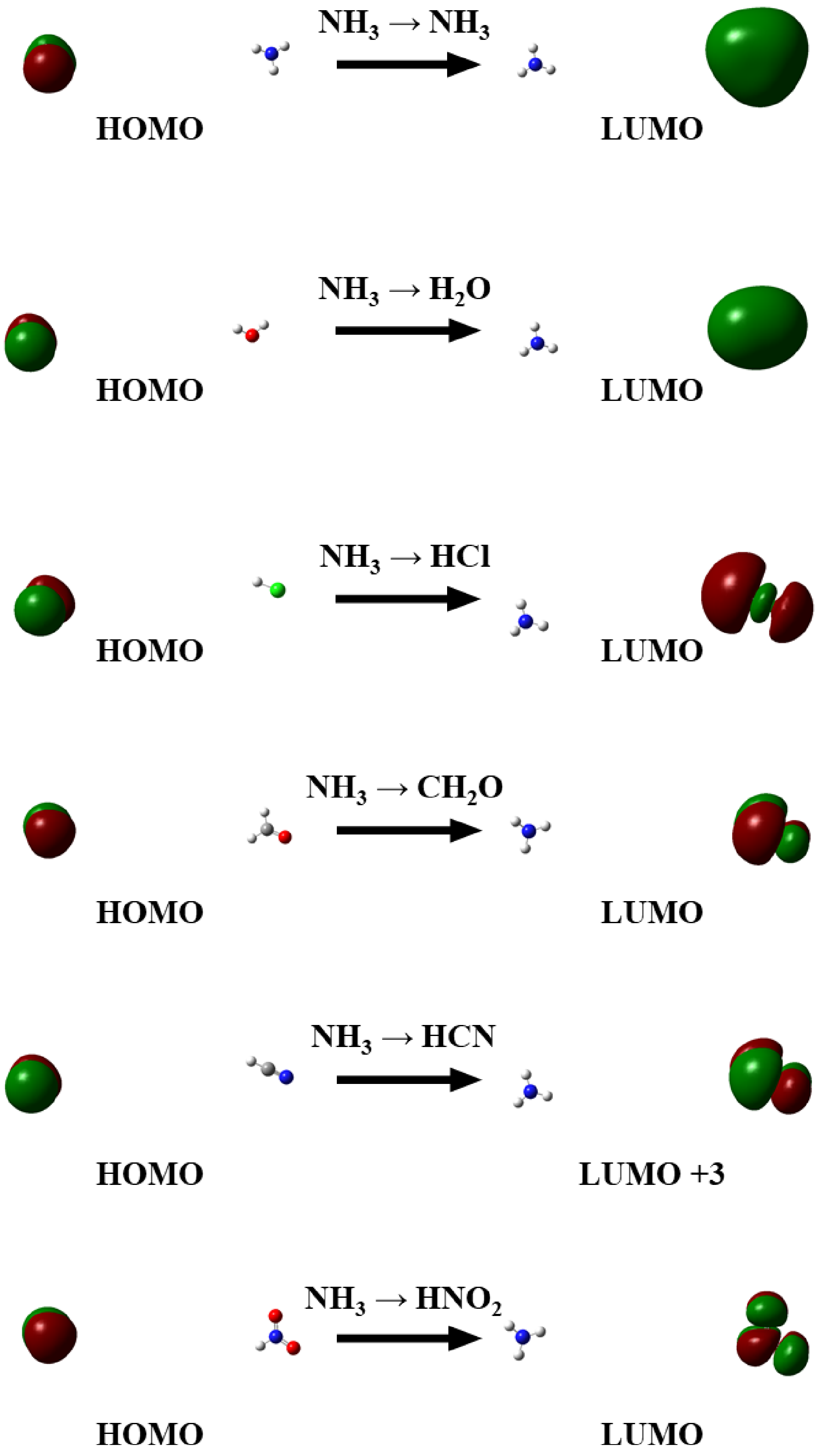


**Figure S14.** MO shapes of intermolecular CT excitations from $NH_3$ to various electron acceptors with $n$=5 calculated using HF.

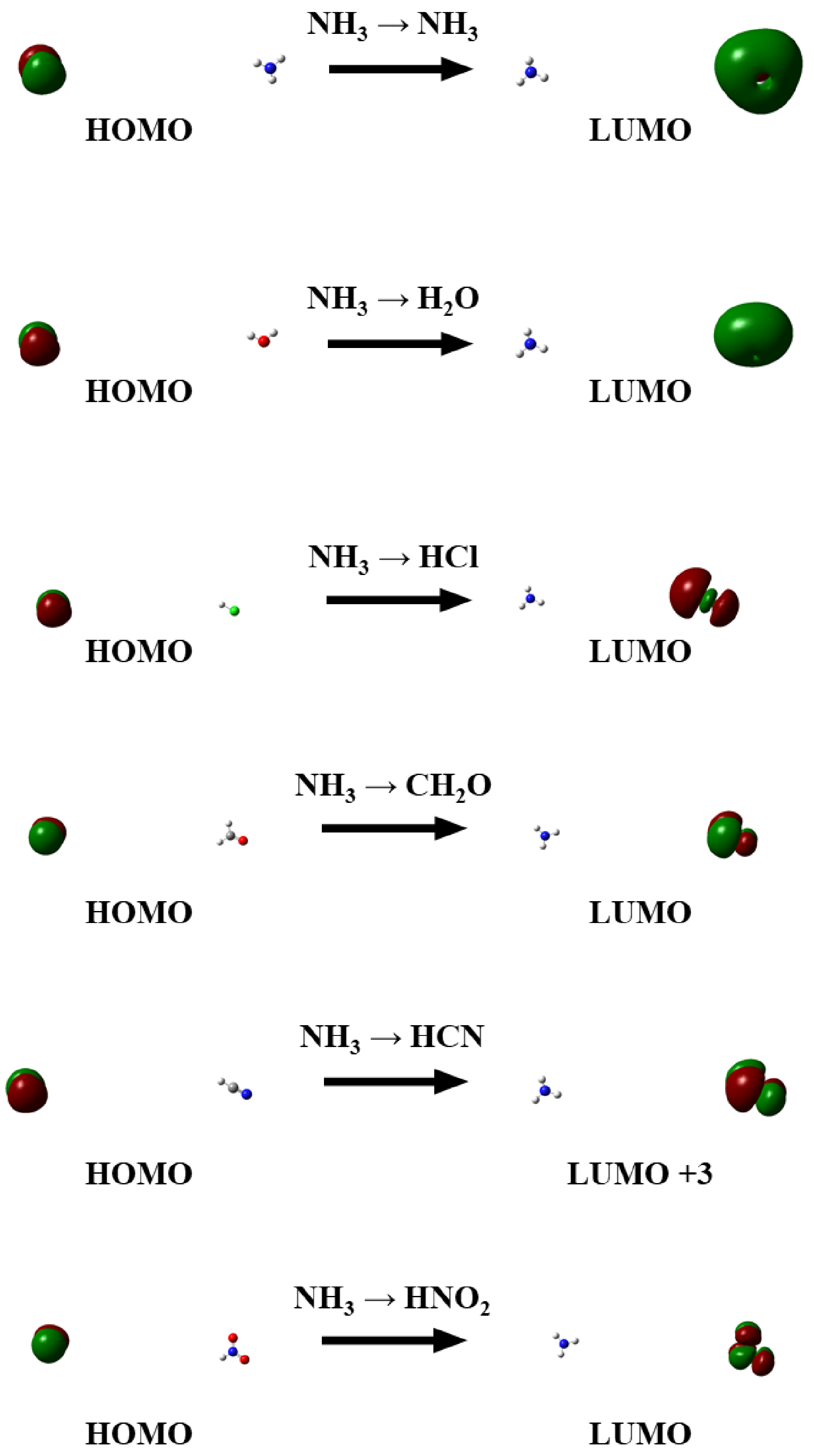


**Figure S15.** MO shapes of intermolecular CT excitations from $NH_3$ to various electron acceptors with *n*=6 calculated using HF.

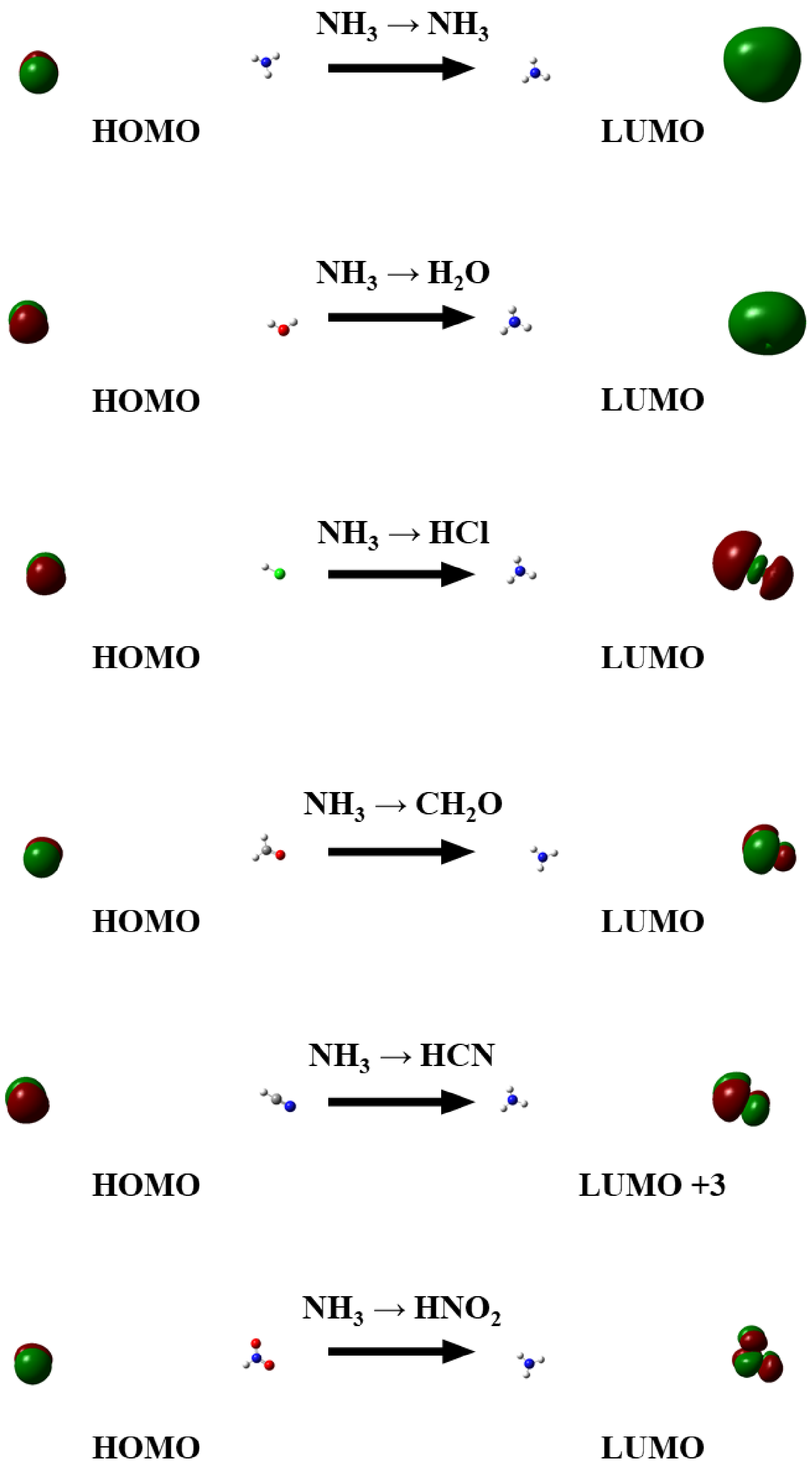


**Figure S16.** MO shapes of intermolecular CT excitations from $NH_3$ to various electron acceptors with $n$=7 calculated using HF.

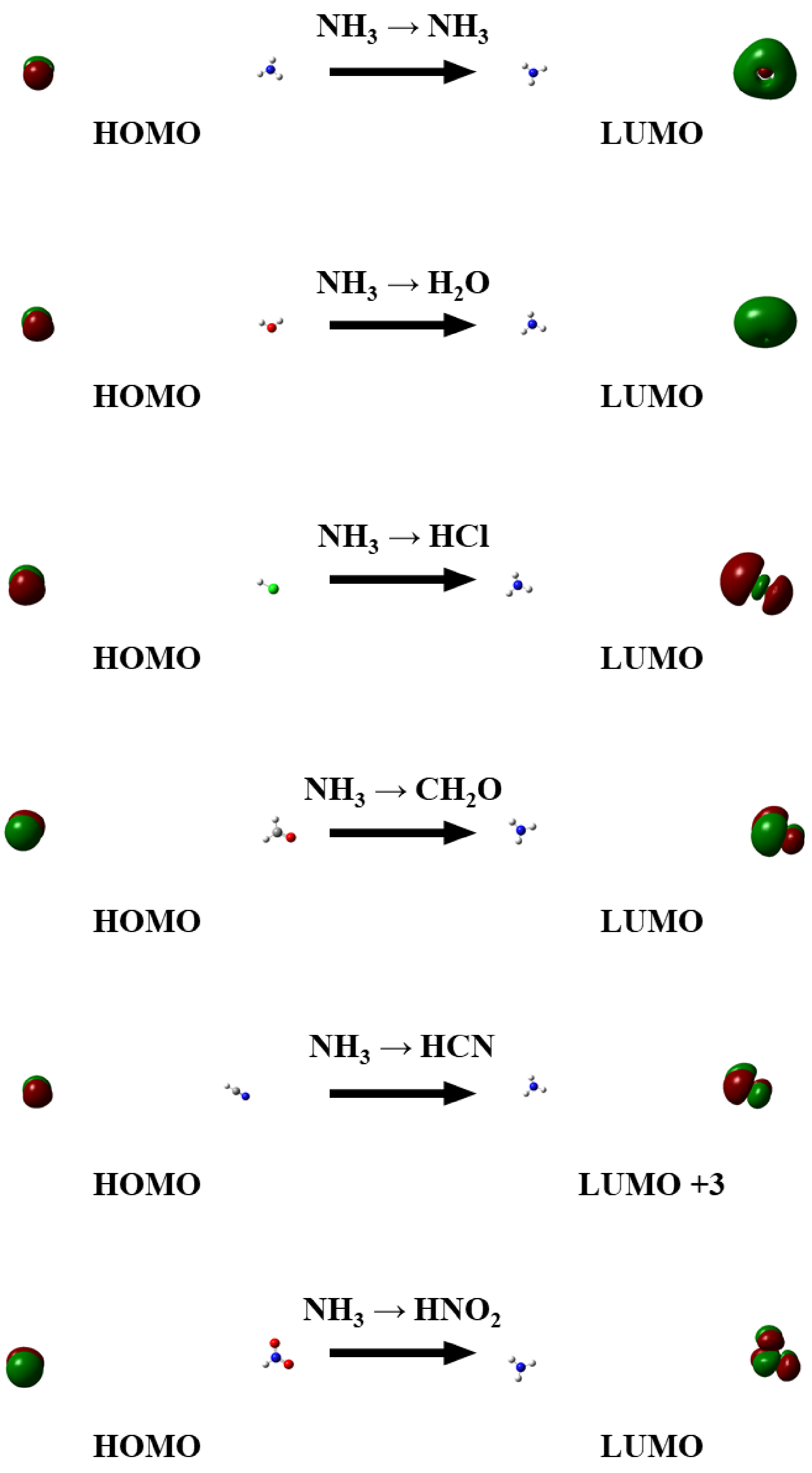


**Figure S17.** MO shapes of intermolecular CT excitations from $NH_3$ to various electron acceptors with *n*=8 calculated using HF.
